# In silico analysis of the cyclophilin repertoire of apicomplexan parasites

**DOI:** 10.1186/1756-3305-2-27

**Published:** 2009-06-25

**Authors:** Jürgen Krücken, Gisela Greif, Georg von Samson-Himmelstjerna

**Affiliations:** 1 Institute for Parasitology, University of Veterinary Medicine Foundation, Bünteweg 17, 30559 Hannover, Germany; 2 Bayer Animal Health GmbH, Research & Development, Leverkusen, Germany

## Abstract

**Background:**

Cyclophilins (Cyps) are peptidyl *cis/trans *isomerases implicated in diverse processes such as protein folding, signal transduction, and RNA processing. They are also candidate drug targets, in particular for the immunosuppressant cyclosporine A. In addition, cyclosporine is known to exhibit anti-parasitic effects on a wide range of organisms including several apicomplexa. In order to obtain new non-immunosuppressive drugs targeting apicomplexan cyclophilins, a profound knowledge of the cyclophilin repertoire of this phylum would be necessary.

**Results:**

BLAST and maximum likelihood analyses identified 16 different cyclophilin subfamilies within the genomes of *Cryptosporidium hominis*, *Toxoplasma gondii*, *Plasmodium falciparum*, *Theileria annulata*, *Theileria parva*, and *Babesia bovis*. In addition to good statistical support from the phylogenetic analysis, these subfamilies are also confirmed by comparison of cyclophilin domain architecture. Within an individual genome, the number of different Cyp genes that could be deduced varies between 7–9 for Cryptosporidia and 14 for *T. gondii*. Many of the putative apicomplexan cyclophilins are predicted to be nuclear proteins, most of them presumably involved in RNA processing.

**Conclusion:**

The genomes of apicomplexa harbor a cyclophilin repertoire that is at least as complex as that of most fungi. The identification of Cyp subfamilies that are specific for lower eukaryotes, apicomplexa, or even the genus Plasmodium is of particular interest since these subfamilies are not present in host cells and might therefore represent attractive drug targets.

## Background

Cyclophilins (Cyps) represent an ancient protein family with peptidyl-prolyl *cis/trans *isomerase (PPIase), also called rotamase, activity (EC 5.2.1.8) that can be found in archea, prokaryotes and eukaryotes [1,2]. PPIases catalyze the *cis/trans *isomerization of peptide bonds preceding a prolyl residue in polypeptides. Although ribosomes synthesize proteins with peptidyl-prolyl bonds in the lower energy trans state, about 5–7% of these bonds are estimated to occur in the unfavorable *cis *conformation [3]. PPIases are thought to be important for establishing this conformation during protein folding or refolding after transport of proteins into organelles [1] by stabilizing the *cis/trans *transition state [4]. Moreover, some Cyps possess chaperone activity that is independent from their PPIase activity [5]. Many Cyps are able to bind the widely used immunosuppressant cyclosporin A (CsA) that on one hand inhibits their PPIase activity but on the other hand results in a gain of function phenotype due to binding of Cyp/CsA complexes to calcineurin-like phosphatases resulting in inhibition of phosphatase activity. In mammalian T cells, inhibition of calcineurin by Cyp/CsA complexes after T cell receptor stimulation prevents transcription of the autocrine growth factor IL-2 resulting in immunosuppression.

In addition to cyclophilins, two also widely spread but structurally unrelated protein families, FK506-binding proteins (FKBP) and parvulins, also exhibit PPIase activity [3].

Eukaryotic genomes usually encode several Cyps. Small Cyps containing only a single Cyp domain are present along with larger multi-domain proteins containing a Cyp domain in addition to one or several unrelated domains. For instance, the genome of the fission yeast *Schizosaccharomyces pombe *contains four single domain Cyps – including *Sp*Cyp4 which has a signal peptide and can be found in the ER – and five multi domain Cyps [6]. Two (*Encephalitozoon cuniculi*), eight (*Saccharomyces cerevisiae*) and 17 (*Rhizopus oryzae*) Cyps could be identified [7-9]*e.g*. in the genomes of representative microsporidia and fungi. Despite their ubiquitous expression and high evolutionary conservation, convincing evidence for the importance of Cyps for cellular homeostasis is largely missing. In *S. cerevisae*, for instance, none of the eight Cyps is essential, and even a mutant lacking all eight Cyps and four FKBPs simultaneously has only a subtle phenotype [10].

Parasite Cyps have received increasing attention in recent years (see [11] for review) in particular because CsA has not only immunosuppressive but also anti-parasitic activity as already demonstrated in 1981 for schistosoma and murine malaria infections [12,13]. Since then, anti-parasitic activity of CsA has been demonstrated for numerous protozoan and helminth parasites [11,14]. Because the anti-parasitic effects of CsA can be superimposed *in vivo *by its immunosuppressive action, treatment of infected animals with CsA may either result in resolution/amelioration or aggravation of the clinical course [11]. However, the development of non-immonosuppressive CsA analogs that retain anti-parasitic activity shows that parasite Cyps may well be attractive drug targets [15].

Since the discovery of CsA sensitivity of *Plasmodium chabaudi *and *Plasmodium berghei *[13], development of several other apicomplexa has been described to be inhibitable by CsA including *Plasmodium falciparum *[16], *Toxoplasma gondii *[17], *Eimeria tenella *[18], *Eimeria vermiformis*, *Eimeria mitis *[19], and *Cryptosporidium parvum *[20]. In contrast, *Theileria annulata *schizonts appear to be unaffected by CsA though the drug inhibits proliferation of *Theileria*-transformed lymphocytes – presumably by acting on host cell Cyps [21].

Despite the long time since discovery of CsA effects on these important parasites, current knowledge about the anti-parasitic mechanisms of CsA is rather limited. For *P. falciparum*, two major small cytosolic Cyps and their inhibition by CsA and CsA derivates have been described [22-24]. Inhibition of *P. falciparum *calcineurin by a complex of CsA and *Pf*Cyp19 (= PfCyp19A in reference [25]) has also been demonstrated biochemically [26]. Using sequence analysis of highly CsA-resistant mutant lines of *P. falciparum*, Kumar *et al*. [25] could show that point mutations in the regulatory or the catalytic subunit of calcineurin or in *Pf*Cyp19 or *Pf*Cyp21.7 (= *Pf*Cyp19B) are sufficient to induce CsA resistance. In contrast, no mutations in the *Pf*Cyp24.6 (= *Pf*Cyp24) gene were identified. However, since CsA resistance in five out of nine mutant lines was not associated with changes in the sequence of any of these four genes, additional gene products can be expected to be involved in CsA action in *P. falciparum*. The situation is even more complicated by the fact that at least certain non-immunosuppressive CsA derivates have been shown to have profound anti-parasitic effects possibly by acting on ABC transporters of the multi-drug-resistance protein family in *T. gondii *and *P. faciparum *[15,27].

In addition to their role as putative drug targets, cyclophilins of apicomplexan parasites are also interesting from an evolutionary point of view, since a novel group of dual family PPIases has been recently described for *T. gondii*, which contain both a Cyp and an FKBP domain in the same protein [28]. Such FCBPs (FK506- and cyclosporin-binding proteins) appear to be present in the genomes of archae- and eubacteria as well [5], and the phylogenetic relationship of apicomplexan FCBP with such non-eukaryotic enzymes remains to be addressed.

Up to now, research on apicomplexan Cyps has focused on small, abundant single-domain Cyps. Only recently, a multi-domain WD40 repeat containing Cyp has been described for *E. tenella *[29]. The progress in genome sequencing projects for several apicomplexan parasites allows now for systematic searches for cyclophilins and will presumably bring the multi-domain Cyps more into the focus of research. This work is aimed to provide a framework for such analysis by identifying and comparing the cyclophilin repertoire of the important apicomplexan pathogens *T. gondii*, *P. falciparum*, *Theileria parva*, *T. annulata*, *Babesia bovis*, and *Cryptosporidium hominis*.

## Results and discussion

### Identification of open reading frames for Cyps

In order to identify open reading frames (ORFs) encoding putative Cyps, BLAST and TBLASTn analyses against GenBank^®^, genomic sequence data and deduced coding sequences were performed. The Cyp proteins deduced from *T. gondii*, *P. falciparum*, *T. annulata*, *T. parvum*, *B. bovis*, and *C. hominis *are listed in Tables 1, 2, 3, 4, 5, 6, respectively. Two putative Cyps from *Cryptosporidium muris *were included in the analysis, because the orthologous Cyps could not be identified in the genome of *C. hominis*. Moreover, two Cyps deduced from the *Plasmodium yoelii *genome were included as the corresponding *P. falciparum *are quite unusual. Table S1 – in Additional file 1 in the supplemental online material – lists all Cyp proteins encoded in the genomes of *S. pombe *and *Homo sapiens *that were used for comparison with the apicomplexan Cyp repertoire.

**Table 1:** *Cryptosporidium *cyclophilin proteins.

Name^a^	Accession-no.^b^	Amino acids	MW^c ^(kDa)	Motifs/Domains^d^
*Ch*Cyp17.9	3413232	167	17.9	Cyp
*Ch*Cyp18.4	3415531	172	18.4	Cyp
*Ch*Cyp18.9	3415323	169	18.9	Cyp
*Ch*Cyp21.2	3412170	189	21.2	Cyp
*Ch*Cyp22.9	3413640	210	22.9	SP, Cyp
*Ch*Cyp34.5	3411992	302	34.5	Cyp, RRM
*Cm*Cyp44.6	6997393	391	44.6	Cyp
*Cm*Cyp48.8	6997190	417	48.8	SYF2, Cyp
*Ch*Cyp88.9	3413617	77.4	88.9	WD40, Cyp

^a ^Deduced from the genome of *Cryptosporidium hominis *(*Ch*Cyp) or *Cryptosporidium muris *(*Cm*Cyp).

^b^Accession number in Entrez Gene.

^c^MW, molecular weight.

^d^Cyp, cyclophiln domain; SP, signal peptide; RRM, RNA recognition motif; SYF2, SYF2 splicing factor; WD40, WD40 repeat; FKBP, FK506-binding domain; TRP, Tetratricopeptide repeat.

**Table 2:** *Toxoplasma gondii *cyclophilin proteins.

Name	Accession-no.	Amino acids	MW^a ^(kDa)	Motifs/Domains^b^
*Tg*Cyp18.7^e^	EEA99351^c^	163	18.7	Cyp
*Tg*Cyp18.8	EEB00823^c^	172	18.8	Cyp
*Tg*Cyp19.6	AAA17997^c^	179	19.6	SP, Cyp
*Tg*Cyp21	EEA98581^c^	195	21.0	Cyp
*Tg*Cyp21.7	EEB01220^c^	197	21.7	Cyp
*Tg*Cyp23	EEB02074^c^	211	23.0	Cyp
*Tg*Cyp31.8^e^	TGGT1_052840^d^	283	31.8	Mito, Cyp
TgCyp36.7^f^	ORF^f^	335	36.7	Cyp
*Tg*Cyp38.2	EEB00661^c^	348	38.2	Cyp
*Tg*Cyp64.5	EEA99592^c^	575	64.5	Cyp
*Tg*Cyp66.2	EEB00778^c^	587	66.2	Cyp, SYF2
*Tg*Cyp66.3^g^	EEA99267^c^	592	66.3	Cyp^e^, RRM
*Tg*Cyp72.9	EEB03226^c^	612	72.9	RING, Cyp
*Tg*Cyp86	EEB02781^c^	764	86	WD40, Cyp
*Tg*FCBP57.3	AAX51680^c^	521	57.3	FKBP, TRP, Cyp

^a^MW, molecular weight.

^b^Cyp, cyclophiln domain; SP, signal peptide; Mito, mitochondrial targeting sequence; SYF2, SYF2 splicing factor; RRM, RNA recognition motif; RING, RING finger domain; WD40, WD40 repeat; FKBP, FK506-binding domain; TRP, Tetratricopeptide repeat.

^c, d^Accession number in Entrez Protein and ToxoDB, respectively.

^e^*Tg*Cyp18.7 in Entrez Protein contains only an NH_2_-terminally truncated Cyp domain. In all further analyses, *Tg*Cyp31.8 was used instead. This deduced protein contains the same COOH-terminus but additional NH_2_-terminal sequences.

^f^*Tg*Cyp36.7 was deduced from the *T. gondii *VEG genome sequence, chromosome VIIa, between position 1860418 and 1864189.

^g^*Tg*Cyp66.3 contains a protein containing a RNA recognition motif. However, the Cyp domain appears to be disrupted, maybe due to misprediction of the ORF.

**Table 3:** *Plasmodium *cyclophilin proteins.

Name	Accession-no.^a^	Amino acids	MW^b ^(kDa)	Motifs/Domains^c^
*Pf*Cyp18.6	810717	167	18.6	Cyp
*Pf*Cyp19	814534	171	19	Cyp
*Pf*Cyp21.7	810711	195	21.7	SP, Cyp
*Pf*Cyp23.2	813100	204	23.2	Cyp
*Pf*Cyp24.9	2655320	217	24.9	Cyp
*Pf*Cyp26.4	811077	226	24.6	Cyp
*Pf*Cyp32.3	811200	280	32.3	Mito, Cyp
*Pf*Cyp51.8	811805	440	51.8	Cyp
*Pf*Cyp72.5	813578	609	72.5	Cyp
*Pf*Cyp80.9	2655327	677	80.9	Cyp, SYF2
*Pf*Cyp87	812836	747	87	WD40, Cyp
*Py*Cyp69.8	3830381	587	69.8	Cyp
*Py*Cyp74	3791457	621	74	Cyp, SYF2

^a ^Accession number in Entrez Gene.

^b ^MW, molecular weight.

^c ^Cyp, cyclophilin domain; SP, signal peptide; Mito, mitochondrial targeting sequence; SYF2, SYF2 splicing factor; WD40, WD40 repeat; FKBP, FK506-binding domain; TRP, Tetratricopeptide repeat.

**Table 4:** *Theileria parva *cyclophilin proteins.

Name	Accession-no.^a^	Amino acids	MW^b ^(kDa)	Motifs/Domains^c^
*Tp*Cyp18.4	3500351	164	18.4	Cyp
*Tp*Cyp20.3	3502670	175	20.3	Cyp
*Tp*Cyp21.4	3501804	196	21.4	SP, Cyp
*Tp*Cyp24.4	3502715	217	24.4	Cyp
*Tp*Cyp24.5	3503253	216	24.5	Mito, Cyp
*Tp*Cyp25.5	3502505	227	25.5	AP, Cyp
*Tp*Cyp51.4	3501424	445	51.4	Cyp
*Tp*Cyp58.8	3501575	517	58.8	RING, Cyp
*Tp*Cyp59.8^d^	3500432	527	59.8	Cyp
*Tp*Cyp61.3	3503208	539	61.3	WD40, Cyp
*Tp*FCBP51.4	3861988	460	51.4	FKBP, TRP, Cyp

^a ^Accession number in Entrez Gene.

^b ^MW, molecular weight.

^c ^Cyp, cyclophiln domain; SP, signal peptide; Mito, mitochondrial targeting sequence; AP, apicoplast targeting sequence; RING, RING finger domain; WD40, WD40 repeat; FKBP, FK506-binding domain; TRP, Tetratricopeptide repeat.

^d ^NH_2_-terminus extended according to similarity with *Ta*Cyp63.

**Table 5:** *Theileria annulata *cyclophilin proteins.

Name	Accession-no.^a^	Amino acids	MW^b ^(kDa)	Motifs/Domains^c^
*Ta*Cyp18.3^d^	3865382	164	18.3	Cyp
*Ta*Cyp21.6	3862085	196	21.6	SP, Cyp
*Ta*Cyp22.8	3863397	205	22.8	Cyp
*Ta*Cyp24.8	3864489	220	24.8	Mito, Cyp
*Ta*Cyp25.7	3864051	228	25.7	AP, Cyp
*Ta*Cyp48.8	3861733	426	48.8	Cyp
*Ta*Cyp58.8	3861717	515	515	RING, Cyp
*Ta*Cyp63	3864616	553	63.0	Cyp
*Ta*Cyp70	3863563	613	70	WD40, Cyp
*Ta*FCBP51.3^e^	3861988	459	51.3	FKBP, TRP, Cyp

^a ^Accession number in Entrez Gene.

^b ^MW, molecular weight.

^c ^Cyp, cyclophiln domain; SP, signal peptide; Mito, mitochondrial targeting sequence; AP, apicoplast targeting sequence; RING, RING finger domain; WD40, WD40 repeat; FKBP, FK506-binding domain; TRP, Tetratricopeptide repeat.

^d^Residues 44–54 deleted to restore similarity to all other cyclophilins. These eleven amino acids presumably represent an intron that was not recognized by the prediction algorithm.

^e ^NH_2_-terminus extended according to similarity with *Tp*Cyp51.4.

**Table 6:** *Babesia bovis *cyclophilin proteins.

Name	Accession-no.^a^	Amino acids	MW^b ^(kDa)	Motifs/Domains^c^
*Bb*Cyp19.2	5477772	175	19.2	Cyp
*Bb*Cyp21.1	5480474	195	21.1	SP, Cyp
*Bb*Cyp21.9	5479361	200	21.9	Cyp
*Bb*Cyp23.7	5478663	217	23.7	SP, Cyp
*Bb*Cyp26.9	5477314	242	26.9	Mito, Cyp
*Bb*Cyp28.6	5477723	248	28.6	Cyp
*Bb*Cyp39.4	5478386	354	39.4	Cyp
*Bb*Cyp57.3	5478847	508	57.3	Cyp, SYF2
*Bb*Cyp59.4	5478371	524	59.4	U box, Cyp
*Bb*Cyp65.8	5479111	589	65.8	WD40, Cyp
*Bb*FCBP51.1	5476893	460	51.1	FKBP, TRP, Cyp

^a ^Accession number in Entrez Gene.

^b ^MW, molecular weight.

^c ^Cyp, cyclophiln domain; SP, signal peptide; Mito, mitochondrial targeting sequence; SYF2, SYF2 splicing factor; RING, RING finger domain; WD40, WD40 repeat; FKBP, FK506-binding domain; TRP, Tetratricopeptide repeat.

The number of putative Cyp genes identified per genome ranges from 7 to 9 for *C. hominis *(whether or not orthologs for *Cm*Cyp44.6 and *Cm*Cyp48.8 are assumed to be present in *C. hominis*) to 14 for *T. gondii*, while the genomes of all four haemosporidia exhibit an intermediate number of 11 putative Cyps per genome. For *T. annulata*, an ortholog to *Tp*Cyp20.3 appears to be present on chromosome 1, however, its complete sequence could not be deduced from the genome data. Therefore, Table 2 lists only 10 Cyps for this organism although 11 Cyps are expected to be present. The number of Cyps in apicomplexan genomes is very similar to the 6 to 11 Cyp genes in the genomes of most fungi although it should be mentioned that there are fungi with extreme low (2 Cyps in the microsporidium *Encephalitozoon cuniculi*) and extreme high (16 Cyps in *Rhizopus oryzae*) numbers of Cyp genes [9]. An extremely high number of 19 Cyp genes per genome can also be found in the kinetoplastid protozoan parasite *Trypanosoma cruzi *[30]. Similar extremes cannot be found in the genomes of the currently sequenced apicomplexa.

### Phylogenetic relationship of Cyp domains

**Figure 1:**
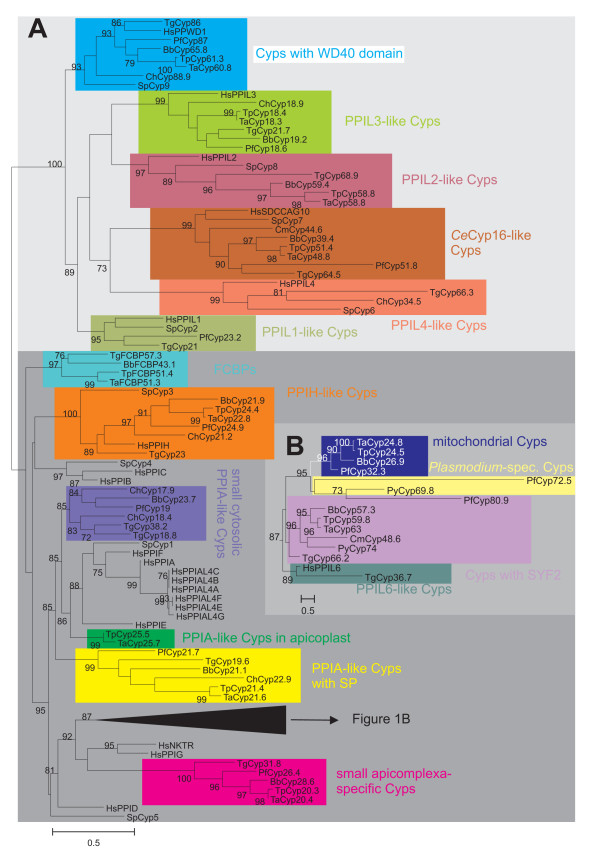
**Unrooted phylogram representing evolutionary relationship between apicomplexan Cyps**. (A) Sequences of putative Cyp domains were aligned using ClustalW2 and an unrooted maximum likelihood tree was calculated using PhyML [34]. For comparison, the human and fission yeast Cyp repertoires were included in the analysis. Statistical support of nodes calculated as likelihood ratios is indicated for those nodes with at least 70% support. Cyp subfamilies as revealed by phylogenetic analysis and domain architecture are highlighted by different colors. The dark gray background in the lower part of the figures marks Cyp subfamilies containing Cyp_ABH type or related domains. (B) Subtree from phylogram presented only compressed in (A). The scale bars represent 0.5 substitutions per amino acid position. *Ch*, *C. hominis*; *Tg*, *T. gondii*; *Pf*, *P. falciparum*; *Py*, *Plasmodium yoelii*; *Bb*, *B. bovis*; *Ta*, *T. annulata*; *Tp*, *T. parva*; *Hs*, *Homo sapiens*; *Sp*, *Schizosaccharomyces pombe*. The identity of individual protein sequences used for analyses can be obtained from Tables 1–6 and Table S1 in Additional file 1.

In order to identify subfamilies within the Cyp repertoire and to analyze their phylogenetic relationship, the putative Cyp domains as identified by CD-BLAST [31,32] were aligned by ClustalW2 [33]. Maximum likelihood analysis with PhyML [34] was used to calculate an unrooted tree shown in Figure 1. Statistical support values at the branches are calculated by a likelihood ratio test which produces values similar but not identical to those obtained by bootstrapping [34].

Although it may be assumed that small Cyps containing only a single Cyp domain have been present early in evolution before occurrence of Cyps with one or more additional domains, it is not possible to unequivocally identify a "primitive" Cyp protein subfamily in the apicomplexa from which all other subfamilies have derived, since there are several single- and multi-domain Cyps in the genomes of all protists analyzed so far. Obviously, many subfamilies of Cyps have already evolved before spread of the major lines of eukaryotic evolution.

According to their phylogenetic relationship, 16 different Cyp protein subfamilies were defined here (Figure 1) – many of them well known from other eukaryotes. All these subfamilies exhibit a statistical support in the likelihood ratio test implemented in PhyML of at least 85% and all families containing Cyps with multiple domains are also supported by their domain architecture. The only exception is the subfamily containing putative Cyps with a so-called SYF2 domain, a domain first described in the yeast splicing factor SYF2 [35]. One of these putative SYF2-containing Cyps, i.e. *Pf*Cyp80.9, has a very divergent sequence that does not fall into the same PhyML-deduced group as the other subfamily members (Figure 1B). The corresponding protein deduced from *P. yoelii *(*Py*Cyp74) was therefore also included and the latter is apparently an ortholog to the SYF2 Cyps of other apicomplexa. Since the subfamily of Cyps with SYF2 is strongly supported by domain architecture and all *Plasmodium *species but *P. falciparum *posses putative SYF2 Cyps with high similarity to *Py*Cyp74, it appears that the putative *Pf*Cyp80.9 was either not predicted correctly or has undergone dramatic alterations after separation of *P. falciparum *from *P. vivax *and the rhodent malaria species. Instead of clustering with other SYF2 Cyps, *Pf*Cyp80.9 forms a group together with a group of large putative Cyps that can only be identified in the genus *Plasmodium *(*Plasmodium*-spec. Cyps), represented in Figure 1 by *Pf*Cyp72.9 and *Py*Cyp69.8.

The phylogram in Figure 1 also indicates the presence of two major groups of Cyps depending on whether they contain a Cyp domain related to the Cyp_ABH subtype (CD database accession number [cd01926], drawn on dark gray background in the lower half of Figure 1A) or any of the non Cyp_ABH-like domains (on light gray background in the upper half of Figure 1A). Within the Cyp_ABH group, it is noteworthy that several important groups of well-known Cyps are absent from apicomplexan genomes whereas there are new Cyp subfamilies that appear to be specific for apicomplexa. On one hand, there are apparently no orthologs of *Hs*PPIB or *Hs*PPIC (both involved in protein folding in the secretory pathway [8]), PPID (function in mitochondrial permeability transition during cell death responses [36,37]), and PPIG (involved in splicing [38]). On the other hand, there are several Cyp subfamilies that are specific at least for lower eukaryotes or even for apicomplexa but do not have orthologs in their mammalian hosts and might therefore be promising drug targets in the future. This includes in particular mitochondrial Cyps, Cyps with SYF2, Cyps with signal peptide, and a group of small, presumably cytosolic Cyps specific for apicomplexa.

The following sections will describe genomic organization and protein domain architecture of these subfamilies beginning with the Cyp_ABH-containing proteins. The different subfamilies will be described in the same order in which they are presented in the phylogenetic tree in Fig. 1.

### PPIA-like small cytoplasmic Cyps and apicoplast Cyps

**Figure 2:**
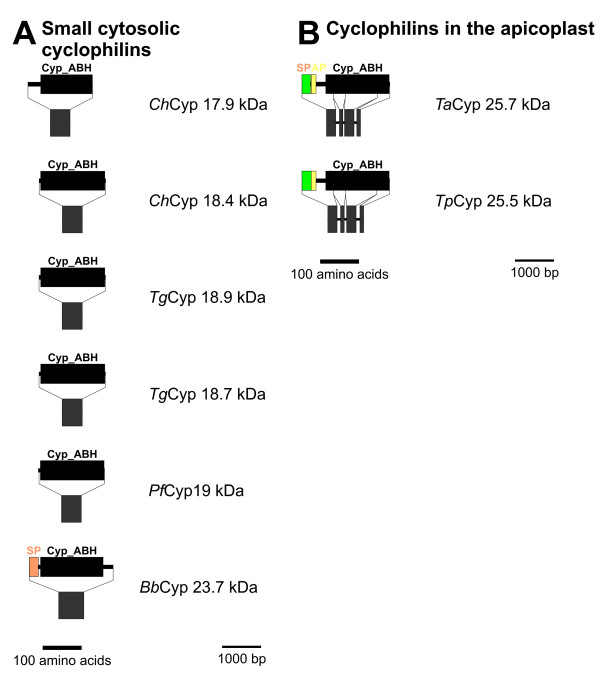
**Domain architecture and genomic organization of PPIA-like Cyps**. (A) PPIA-like cytosolic Cyps. For each Cyp, proteins domains are shown in the upper and exon/intron structure in the lower panel. Proteins and genes are presented by lines, domains and exons are highlighted by boxes. Separate scale bars are given for protein and genome scemes. (B) Cyps with apicoplast localization signal. Species are abbreviated as in Fig. 1. Cyp_ABH, ABH-type Cyp domain (CD accession-no.: [cd01926]); SP, signal peptide; AP, apicolast transit signal.

The prototypical Cyps in humans and *S. pombe*, *Hs*PPIA and *Sp*Cyp1, respectively, are closely related as shown in Figure 1. They form a cluster together with additional human paralogs such as PPIE, PPIF and PPIAL4A-G. The corresponding putative Cyps in apicomplexa, *Ch*Cyp17.9, *Bb*Cyp23.7, *Pf*Cyp19, *Ch*Cyp18.4, *Tg*Cyp18.8, and *Tg*Cyp18.9 (Figure 2A), form a related but separate cluster, i.e. *Sp*Cyp1 is significantly more closely related to *Hs*PPIA, *Hs*PPIE, and *Hs*PPIF than to any of the apicomplexan Cyps. *C. hominis *and *T. gondii *encode two distinct putative members of this PPIA-like subfamily in their genomes. Due to their very high expression levels and their cytoplasmic localization, cytoplasmic Cyps containing a Cyp_ABH type domain (CD database accession-no [cd01926]) are considered to be the most important receptors for CsA leading to inactivation of the cytosolic calcineurins [39]. Indeed, mutations in *Pf*Cyp19 appear to be sufficient to confer resistance to CsA to *P. falciparum *[25].

All these small cytosolic Cyps have no introns interrupting their putative ORFs (Figure 2A).

Conspicuously, the putative PPIA-like Cyps of the piroplasms, *Bb*Cyp23.7 (Figure 2A), *Tp*Cyp25.5 and *Ta*Cyp25.7 (Figure 2B), are predicted to contain an NH_2_-terminal signal peptide (SP) for cotranslational transport into the ER and for the latter two the PATS algorithm [40] predicts an apicoplast transit signal (AP) following the SP. Such combinations of SP and AP are typical for proteins which are transported into the apicoplast [41]. *Tp*Cyp25.5 has been described to be cotranslationally transported into dog pancreas rough microsomes in a wheat germ *in vitro *translation system [42] demonstrating that the SP is functionally active in this heterologous system. Since no removal of the signal peptide was detectable in this system, *Tp*Cyp25.5 was proposed to be anchored to the membrane of the ER via an uncleavable signal peptide. However, since transport of proteins to the apicoplast has been shown to require passage through the ER (for review see [41]) and the properties of proteases responsible for removal of signal peptides might be quite different in apicomplexa and mammals, these results do not exclude that *in vivo Tp*Cyp25.5 is transported further from the lumen of the ER into the apicoplast. Moreover, it cannot yet be excluded that the putative *Bb*Cyp23.7 will turn out to be localized in the apicoplast as well since the sequence between the SP and the Cyp domain is long enough to function as an AP. Since the neural network analysis used in PATS has been trained only on AP of proteins from *P. falciparum*, it might well turn out to be less sensitive to functional AP in other apicomplexa such as *B. bovis*. However, *Tp*Cyp25.5 and *Ta*Cyp25.7 appear to be more closely related to *Hs*PPIA and *Sp*Cyp1 than to the other apicomplexan Cyps of this group whereas *Bb*Cyp23.7 clusters together with the clearly cytoplasmic Cyps of other apicomplexa. Whether the position of apicoplast Cyps in the phylogram truly reflects different evolutionary origins from cytoplasmic Cyps or different selective pressures caused by localization in cytoplasm and apicoplast cannot be decided using the current dataset. In addition to the presence of a putative AP, this group also differs from the small cytosolic apicomplexan Cyps – including *Bb*Cyp23.7 – by the presence of three introns within the coding sequence. In order to evaluate whether these Cyps with AP are more closely related to Cyps from plants or algae, BLASTp analyses were performed against protein database entries from dinoflagellates, red, green, and brown algae, green plants, and *Arabidopsis thaliana*. However, highest similarity was always found to cytosolic PPIA-like Cyps and never to Cyps known to be localized in plastids (data not shown). Experimental evidence concerning the localization of *Bb*Cyp23.7, *Ta*Cyp25.7 and *Tp*Cyp25.5 might provide important information on the evolutionary history of these proteins as well, as they should be considered to be monophyletic if all three turn out to be localized in the apicoplast. Moreover, the fact that *Theileria *species do not have a prototypical cyctosolic PPIA-like Cyp might explain why *T. annulata *is resistant to CsA [21].

### PPIA-like Cyps with signal peptide

**Figure 3:**
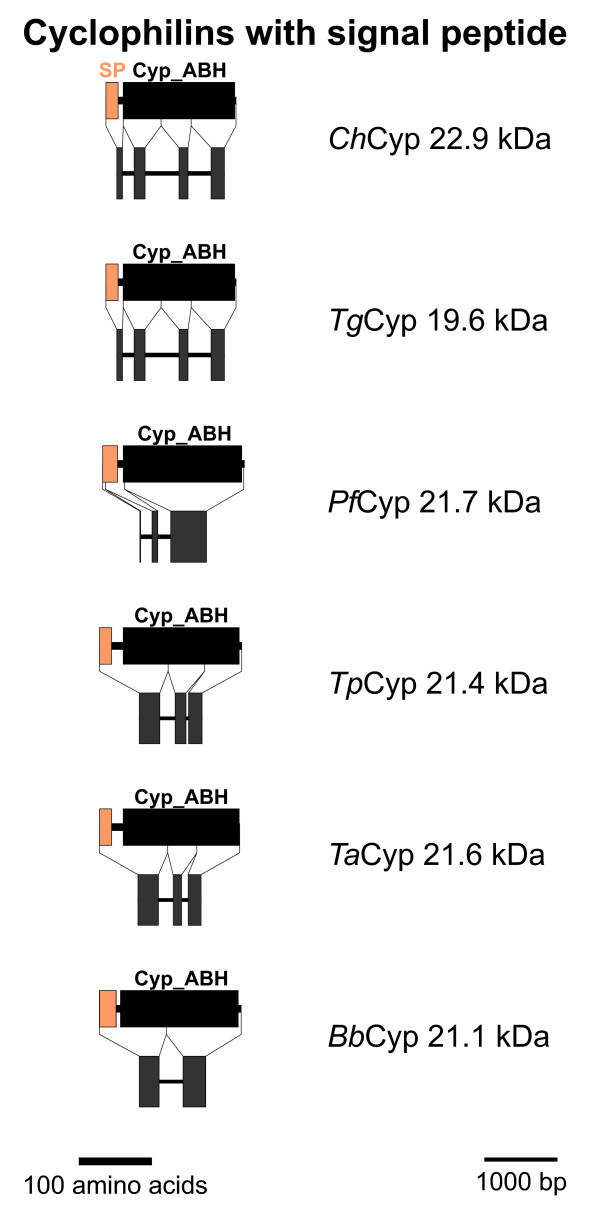
**PPIA-like Cyps with signal peptide**. Domain architecture and genomic organization of Cyps with signal peptide are shown. Species are abbreviated as in Fig. 1. Cyp_ABH, ABH-type Cyp domain (CD accession-no.: [cd01926]); SP, signal peptide.

The dendrogram in Figure 1 reveals a group of putative small Cyps with SP that also contain a Cyp_ABH type domain and are relatively closely related to the PPIA-type Cyps. The domain architecture and genomic organization of these Cyps is schematically presented in Figure 3. One putative member of this Cyp subfamily could be identified in each apicomplexan genome. In contrast to the small cytosolic PPIA-like Cyps, the coding regions of all subfamily members are interrupted by introns. Whereas *C. hominis *and *T. gondii *show a very similar exon/intron structure with 4 introns, the putative genes of both *Theileria *species have only 2 introns – apparently due to fusion of exons 3 and 4 – and after further fusion of exons 1 and 2 only a single intron remains in *B. bovis*. In *Pf*Cyp21.7, loss of introns has resulted in a Cyp domain that is encoded by a single exon. However, a new intron has also appeared within the region encoding the SP, which is encoded by a single exon in the other Cyps of this subfamily.

Cyps within the secretory pathway of other eukaryotes are typically of the PPIB type. The phylogram in Figure 1, however, reveals that the Cyp subfamily with putative ER-localization in apicomplexa does not form any cluster with *Hs*PPIB and *Sp*Cyp4 and is therefore proposed not to represent orthologs of PPIB-like Cyps. Apparently, Cyps in the secretory pathway evolved independently at least twice during evolution of eukaryotic Cyps.

For some members of this Cyp subfamily experimental evidence regarding their expression and function is available. First, the *Pf*Cyp21.7 protein has been shown to be expressed at extremely high levels in blood-stage parasites, constituting up to 0.5% of total cellular protein [24]. Conspicuously, *Pf*Cyp21.7 has been been reported not to be confined to the secretory pathway but to be at least partially present in the cytosol as well [24]. This raises the possibility that this Cyp subfamily might also be able to interact with cytosolic calcineurin-like phosphatases. Indeed, genetic analysis provides evidence that a mutation in *Pf*Cyp21.7 is sufficient to confer resistance to CsA even in the presence of intact *Pf*Cyp19 [25].

Secondly, *Tg*Cyp19.6 has been shown to be secreted by the parasite and to trigger release of IL-12 from host dendritic cells. Moreover, a 19.4 kDa Cyp from *Neospora caninum *belongs to the same orthology group (data not shown). This protein has been described to be secreted by the parasite and to be present in large amounts in culture supernatants of cell infected with *N. caninum *tachyzoites [43]. *Nc*Cyp19.4 from cell culture supernatants was shown to be a very potent inducer of IFNγ production by peripheral blood mononuclear cells and CD4^+ ^T cells. Induction of IFNγ by *Nc*Cyp19.4 could be specifically inhibited by CsA in a dose dependant manner. These results indicate that apicomplexan Cyps with signal peptide are not only involved in protein folding in the secretory pathway but can fulfill important immunomodulatory functions in infected tissues.

### Mitochondrial Cyps

**Figure 4:**
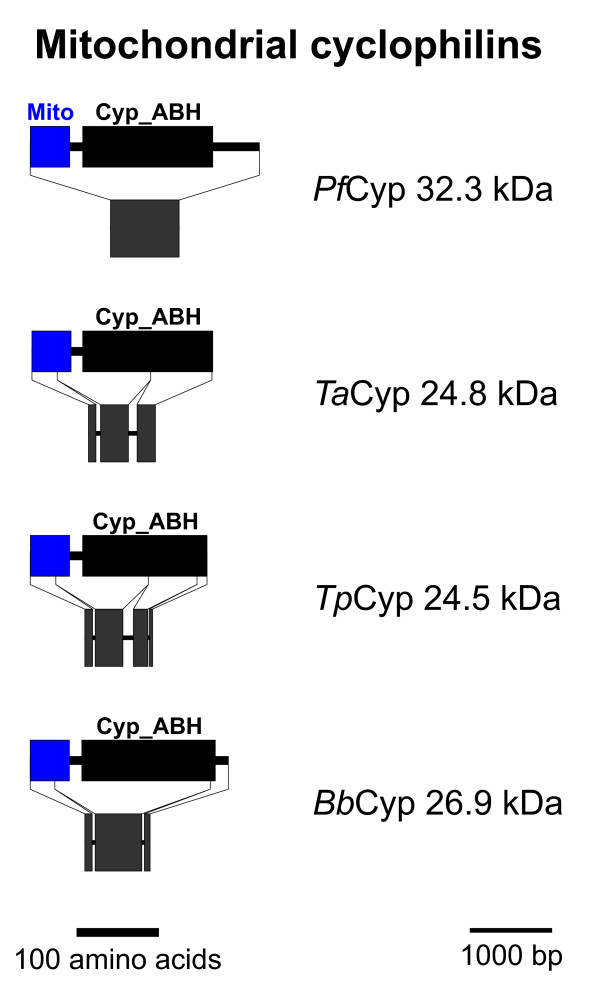
**Mitochondrial Cyps**. Domain architecture and genomic organization of mitochondrial Cyps. Species are abbreviated as in Fig. 1. Cyp_ABH, ABH-type Cyp domain (CD accession-no.: [cd01926]); Mito, mitochondrial localization signal.

Putative Cyps with a mitochondrial localization signal at their NH_2_-terminus are schematically shown in Figure 4. The mitochondrial localization signal and a cleavage site were significantly predicted by MitoProt II [44] for *Ta*Cyp24.8 and *Tp*Cyp24.5. In contrast, cleavage site prediction was not possible for both *Pf*Cyp32.3 and *Bb*Cyp26.9. Nevertheless, MitoProt II predicts a high probability of mitochondrial localization and the algorithm PlasMit [45], which was specifically developed to predict mitochondrial proteins in *Plasmodium*, also suggest a mitochondrial localization of *Pf*Cyp32.3. Putative proteins of this Cyp subfamily could be detected only in the haemosporidia but neither in *T. gondii *nor in *C. hominis*. In *T. gondii*, mitochondrial PPIase activity might be achieved by the putative *Tg*Cyp31.8, a member of the subfamily of apicomplexa-specific Cyps (Figure 6). In contrast to all other members of this group, *Tg*Cyp31.8 is predicted to have an NH_2_-terminal mitochondrial localization signal. However, mitochondrial PPIase activity might also be dispensable in apicomplexan mitochondria as it is completely absent from the genomes of both *C. hominis *and *C. muris*.

The genomic organization differs largely between the different genera with 5 exons in *T. gondii*, 3 or 4 in *T. annulata *and *T. parva*, respectively, and only a single large exon in *P. falciparum *(Figure 4).

### Plasmodium-specific large Cyps

A group of putative Cyp proteins that appears to be present exclusively in *Plasmodium *species is shown in Figure S1 in Additional file 2 in the supplemental online material. In order to demonstrate that these proteins represent a subfamily on their own, the putative *Py*Cyp69.8 was included in the phylogenetic analysis shown in Figure 1. Figure S1 reveals that both *Pf*Cyp72.5 and *Py*Cyp69.8 possess several nuclear localization signals and two coiled-coil domains, which are typically involved in protein-protein interaction. Moreover, PSORT II recognizes an RNA-binding motif typical for components of ribonucleoprotein particles [46] in *Pf*Cyp72.5 further suggesting that this subfamily might somehow be involved in RNA processing.

### Cyps with SYF2 domain

**Figure 5:**
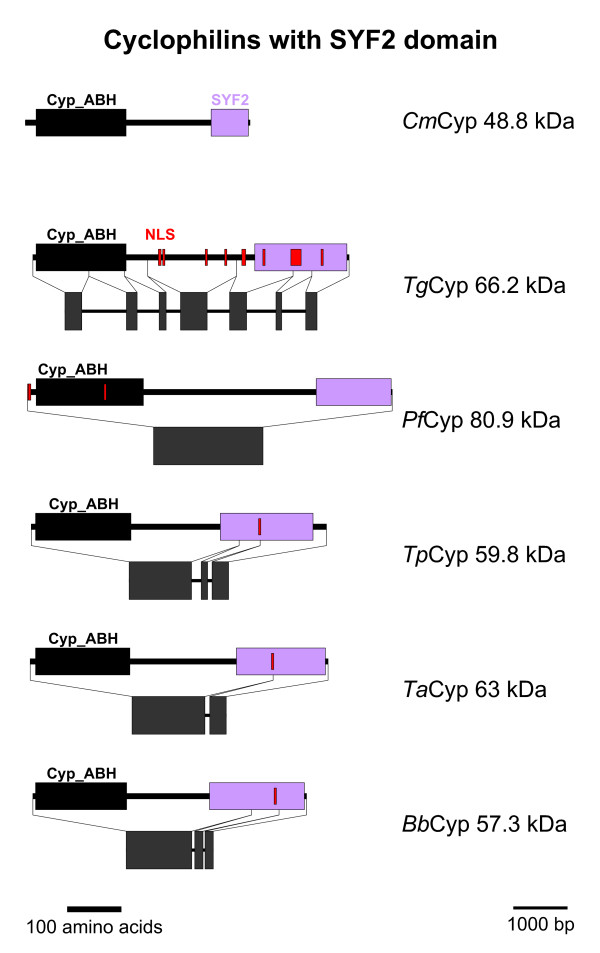
**Cyps with SYF2 domain**. Domain architecture and genomic organization of Cyps with SYF2 domain. Species are abbreviated as in Fig. 1. Cyp_ABH, ABH-type Cyp domain (CD accession-no.: [cd01926]); SYF2, SYF2 splicing factor domain (PFAM accession-no.: [pfam08231]); NLS, nuclear localization signal.

A multi-domain Cyp subfamily within the Cyp_ABH domain group are the predicted Cyps containing an SYF2 domain (PFAM accession-no.: [pfam08231]) (Figures 1 and 5). This subfamily does not form a monophyletic cluster in Figure 1B due to the fact that putative *Pf*Cyp80.9 is quite aberrant and therefore clusters together with the *Plasmodium*-specific Cyps described in the section above. However, since *Pf*Cyp80.9 contains a SYF2 domain and since its ortholog in *P. yoelii*, *Py*Cyp74, is closely related to the other SYF2 domain containing Cyps, this family should nevertheless be considered to be monophyletic.

In the genome of *C. hominis*, a SYF2 Cyp could not be identified, presumably due to incomplete sequence information since a putative orthologues protein is encoded in the genome of *C. muris*, and this sequences was therefore included for further analyses (Figures 1 and 5). The predicted SYF2-Cyps are quite large proteins with predicted molecular weights between 48.8 kDa (*C. muris*) and 80.9 kDa (*P. falciparum*). The Cyp_ABH domain is located in the immediate NH_2_-terminus of the proteins while the SYF2 domain can be found close to the COOH-terminus (Figure 5). The large region between these two defined domains does not exhibit any known sequence features, and homology between individual subfamily members is very low. Only a few scattered amino acids appear to be conserved throughout the subfamily. In accordance with a suspected role in RNA processing, PSORT II predicts at least one nuclear localization signal in all putative SYF2-Cyps but *Cm*Cyp48.8. The latter is also peculiar due to its small size and the presence of only an incomplete SYF2 domain. It must at least be considered that the prediction of the protein coding region of this protein from the genomic sequence is still only partially correct.

**Figure 6:**
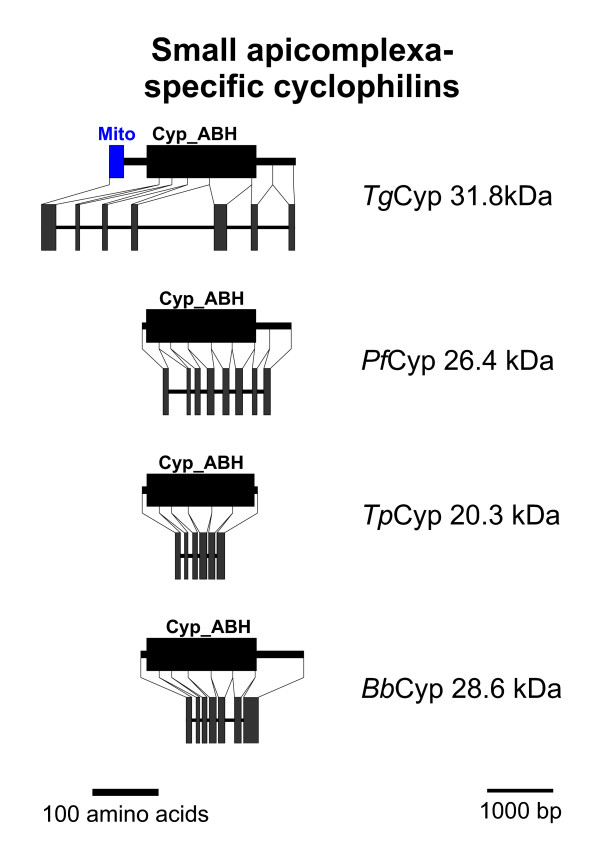
**Small apicomplexa-specific Cyps**. Domain architecture and genomic organization of small apicomplexa-specific Cyps. Species are abbreviated as in Fig. 1. Cyp_ABH, ABH-type Cyp domain (CD accession-no.: [cd01926]); Mito, mitochondrial localization signal.

The genomic organization of SYF2-Cyps is again characterized by progressing loss of introns. While the coding sequence of *Tg*Cyp66.2 is spread across six small exons, there is a large first exon in all other members of the subfamily encoding the Cyp domain, the intervening region and the first part of the SYF2 domain. The remaining sequence is split in two exons in *Tp*Cyp59.8 and *Bb*Cyp57.3 which have further fused in *Ta*Cyp63 to give a two exon structure. Finally, the coding sequence of *Pf*Cyp80.9 is encoded by a single very large exon. For *Cm*Cyp48.8, only parts of the coding sequence could be identified in the available genomic sequences – further challenging the reliability of the predicted protein sequence. Therefore, schematic presentation of the genomic organisation of *Cm*Cyp48.8 is not shown in Figure 6.

SYF2 is a nuclear protein described to be involved in splicing of primary transcripts in the yeast *Saccharomyces cerevisiae *suggesting a contribution of SYF2-Cyps in RNA processing. Cyps with an SYF2 domain have not been identified in higher eukaryotes, however, the CDART tool [47] retrieves three non-apicomplexan putative protein sequences with a similar domain architecture from the ciliates *Tetrahymena thermophila *(accession-no.: [XP_001019212]) and *Paramecium tetraurelium *[XP_001423850] as well as from the primitive green algae *Ostreococcus tauri *(chlorophyta) [CAL53491].

### PPIL6-like Cyp TgCyp36.7

The sequence of *Tg*Cyp36.7 has been predicted by the TwinScan algorithm [48], however, this sequence (TgTwinScan_3870) is no longer available in the predicted ORFs of the *T. gondii *genome. Since this is the only gene prediction from this region of chromosome VII containing the complete Cyp domain, it was nevertheless included in the analyses though the correct prediction of the ORF outside the Cyp domain is quite dubious. The putative *Tg*Cyp36.7 protein (Figure S2 in Additional file 3) is very perculiar in several aspects. First, it does not have orthologues in any of the other apicomplexan genomes (Figure 1). Secondly, despite its relatively large size, the only known protein domain recognized within its sequence is a Cyp domain. Though the latter clusters together with Cyp_ABH domains in Figure 1, it is not recognized as this domain subtype by CD-BLAST but only as general Cyp domain [cd00137]. Thirdly, *Tg*Cyp36.7 and *Hs*PPIL6 form a very significant cluster in the phylogenetic analysis (Figure 1) indicating that they might be orthologs. Indeed, size and domain architecture of both proteins are similar. However, the huge evolutionary distance between *Tg*Cyp37.7 and *Hs*PPIL6 – indicated by the long branch leading to *Tg*Cyp36.7 in the phylogram (Figure 1) – severely questions this hypothesis. Currently, no functional data are available for either PPIL6 or *Tg*Cyp36.7.

### Small apicomplexa-specific Cyps

An additional subfamily of relatively small putative Cyps containing a Cyp_ABH domain can be identified in most apicomplexan genomes with the exception of *C. hominis *and *T. annulata *(Figures 1 and 6). Since there is also no evidence for an ortholog from *C. muris *or *Cryptosporidium parvum *sequences, the conclusion that this subfamily was lost in the genus *Cryptosporidium *appears to be valid. In contrast, BLAST analysis indicates the presence of an orthologous gene on chromosome I of *T. annulata *though the coding sequence could not be completely deduced – maybe due to insufficient sequence quality of the genome sequence. Domain architecture of this Cyp subfamily (Figure 5) reveals that there is a considerably larger heterogeneity than for the two groups described above. First, the putative *Tg*Cyp31.8 sequence contains an additional NH_2_-terminal mitochondrial localization signal as predicted by MitoProtII [44]. Due to the fact that this signal is only observable in a single species and would indicate a significant functional difference to its orthologs in other apicomplexa, careful experimental analyses are needed to compare localization and function of this group of Cyps in different apicomplexa. Secondly, *Tp*Cyp20.3 is very small and consists of little more than a Cyp domain, while *Bb*Cyp28.6, *Pf*Cyp26.4, and *Tg*Cyp31.5 have considerable COOH-terminal extensions. Functional data on this Cyp subfamily are completely missing yet. This group of Cyps has obviously no direct orthologs in mammalian genomes and appears to be specific for apicomplexa. In BLASTp analyses, the most closely related non-apicomplexan Cyps appear to be of plant origin (data not shown). The fact that most of these proteins are predicted to be cytoplasmic and that they have no orthologs in mammalian hosts makes them an attractive target to develop drugs such as non-immunosuppressive CsA derivatives that might specifically target this Cyp subfamily.

### PPIH-like Cyps

**Figure 7:**
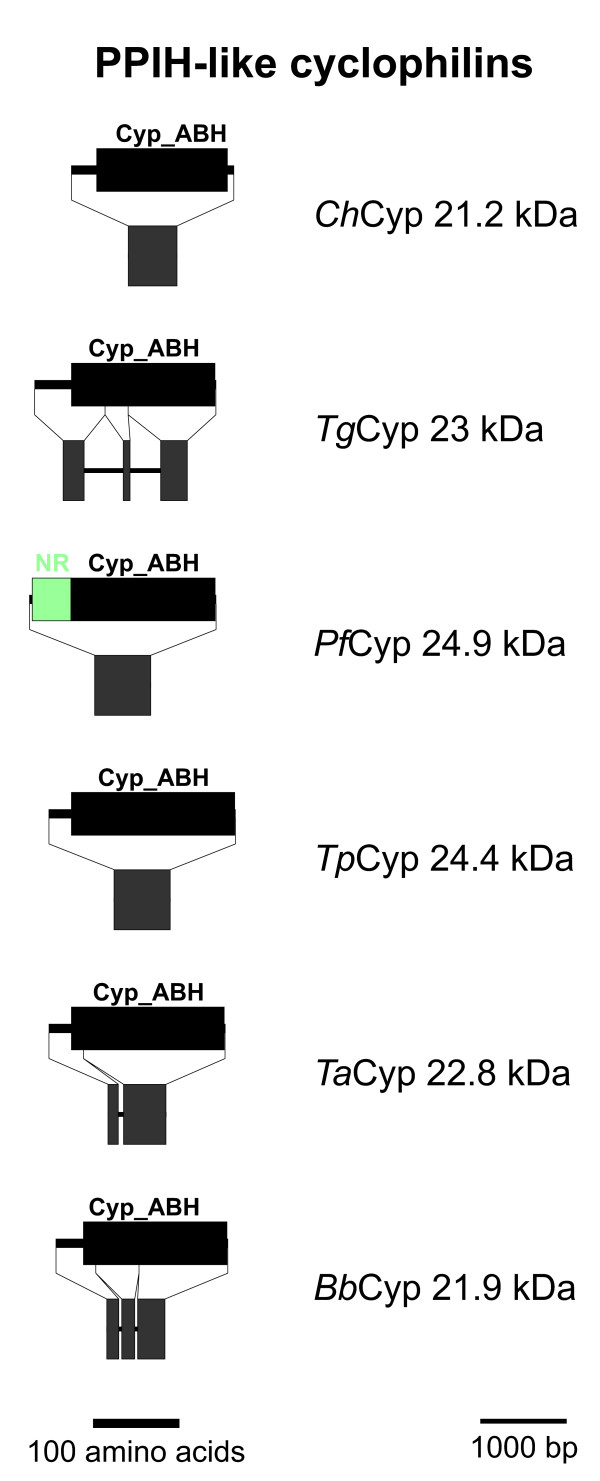
**PPIH-like Cyps**. Domain architecture and genomic organization of PPIH-like Cyps. Species are abbreviated as in Fig. 1. Cyp_ABH, ABH-type Cyp domain (CD accession-no.: [cd01926]); NR-rich, Asn-rich domain.

The PPIH-like Cyps represent another subfamily containing a Cyp_ABH domain that is predicted to be present in all analyzed apicomplexan genomes (Figures 1 and 7). In addition to their Cyp domain, these putative proteins have a short NH_2_-terminal extension which does not contain any recognizable motifs or domains. Only in *Pf*Cyp24.9 this NH_2_-terminal region is characterized by its richness in Asn residues. Though none of the putative apicomplexan PPIH-like Cyps contains any obvious subcellular localization signals, it should be mentioned that their human ortholog has been described to be located in the nucleus and to be associated with the splicing machinery [49,50]. Specifically, *Hs*PPIH is able to interact independently with the factors *Hs*Prp3 and *Hs*Prp4 that both integrate into the U4/U6 di-snRNP particle. The binding-site of *Hs*Prp3 and *Hs*Prp4 for *Hs*PPIH is highly homologous, and binding does not need enzymatic activity of PPIH since it is not impaired by the presence of CsA. PPIH-like Cyps are highly conserved between apicomplexa, fungi and mammals suggesting that the apicomplexan orthologs might carry out similar functions as well.

### FCBP proteins

**Figure 8:**
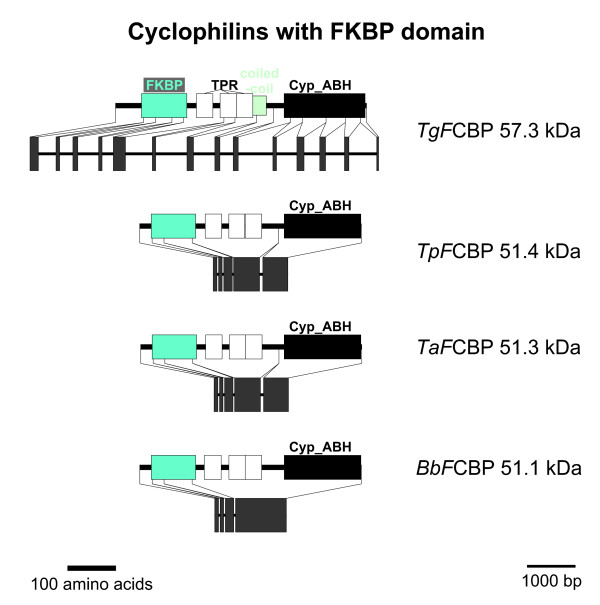
**FCBP proteins**. Domain architecture and genomic organization of FCBPs from apicomplexa. Species are abbreviated as in Fig. 1. Cyp_ABH, ABH-type Cyp domain (CD accession-no.: [cd01926]); FKBP, FK506-binding domain (PFAM accession-no.: [pfam00254]); TPR, Tetratricopeptide repeat (InterProScan accession-no.: [IPR001440]).

The next multi-domain Cyp subfamily to be described here are the recently identified FCBP proteins [28] which contain two phylogenetically unrelated PPIase domains, i.e. an FK506-binding domain (FKBP) at the NH_2_-terminus and a Cyp_ABH type domain in the COOH-terminus (Figure 8). Between these two enzymatic domains, there are three tetratricopeptide repeat domains (TRP) [cd00189] which are typically involved in protein/protein interactions and might contribute to recruitment of specific substrates for FCBP proteins.

Isomerase and chaperone activity have been demonstrated for both PPIase domains of *Tg*FCBP57.3 and the inhibitors FK506 and CsA can suppress activity of the FKBP and Cyp domain, respectively [28]. Moreover, Adams *et al*. [28] could show that only the complex of the FKBP domain with FK506 but not the complex of the Cyp domain with CsA was able to inhibit *T. gondii *calcineurin protein phosphatase activity. Although a weakly synergistic inhibitory effect of FK506 and CsA on parasite growth was noted, this must not necessarily be due to action of *Tg*FCBP57.3 but can also involve any of the other Cyp or FKBP proteins expressed by *T. gondii*. More convincing as a first hint for an important role of FCBPs in the physiology of apicomplexa is the fact that suppression of *Tg*Cyp57.3 expression by RNA interference results in severely decreased incorporation of [^3^H]uracil [28].

In addition to *Tg*FCBP57.3, putative FCBP proteins can be found only in the genomes of *T. parva*, *T. annulata*, and *B. bovis *but not in any of the *Plasmodium *or *Cryptosporidium *species (Figures 1 and 8). In all four apicomplexan FCBP proteins, the enzymatically active domains are separated by TRP repeats. Conspicuously, BLASTp and tBLASTn analyses of protein and nucleic acid databases as well as the CDART tool reveal that putative proteins containing both a Cyp and a FKBP domain are present even in very distantly related organisms such as bacteria [5,28] (see Tables S2 and S3 in Additional file 4 in the supplemental online material for accession-no.). Furthermore, putative FCBP proteins can also be identified in the ciliophora *T. thermophila *and *P. tetraurelia *(Figure S3 in Additional file 5). Since ciliophora and apicomplexa are considered to be phylogenetically related and are usually placed together with dinoflagellates in the infrakingdom alveolata [51], this finding suggests that FCBP proteins were already present in their common ancestors. This hypothesis is also supported by the fact that the deduced FCBPs of ciliophora are also separated by TRP repeats. However, at least the putative *Tt*FCBP131.6 appears to have evolved new or additional functions, since this protein exhibits the presence of an additional (though incomplete) NTPase domain in its very long NH_2_-terminus. Such an NTPase domain can be found neither in its homologs in *Paramecium *nor in the apicomplexan FCBPs. An alternative explanation for the large NH_2_-terminus might be an incorrect prediction of the intron/exon structure resulting in fusion of two adjacent but distinct genes in the database entry. An important argument for the latter hypothesis is the incompleteness and therefore presumably non-functionality of the NTPase domain in the predicted sequence of *Tt*FCBP131.6.

Putative FCBPs can also be identified in the oomycete *Phytophora capsici*, the green algae *O. tauri *(chlorophyta) and in archaebacteria (Figure S3 in Additional file 5). Whereas *Pca*FCBP52.5 also contains a Cyp_ABH domain, the Cyp domains in *O. tauri *CPR7 is truncated and therefore only recognized as Cyp superfamily (accession-no.: [cl00197]). In both predicted archaebacterial FCBPs, CD-BLAST identifies only a Cyp domain without further specification (accession-no.: [cd00317]). In contrast to *Pca*Cyp52.5, neither *Ot*CPR7 nor the archaebacterial FKBPs do contain TRP repeats separating the two PPIase domains (Figure S3). Finally, it should be mentioned that the *Ot*CPR7 sequence might be COOH-terminally truncated since the Cyp domain itself is truncated. In contrast to all other FCBP proteins identified here, *Ot*CPR7 contains an NH_2_-terminal mitochondrial localization signal as predicted with high significance by both PSORT II [52] and MitoProt II.

There are also several putative dual-family immunophilins with an NH_2_-terminal Cyp and a COOH-terminal FKBP domain in proteo- and flavobacteria as well as in spirochaeta (Figure S3). Here, these proteins are called CFBPs, and they do not contain any TRP repeats. All these putative bacterial CFBPs are very similar in size and domain architecture, however, *Borrellia hermsii *CFBP38 has a prokaryotic membrane lipoprotein lipid attachment site (Prosite accession-no.: [PS51257]) at its immediate NH_2_-terminus as identified by InterProScan suggesting that *Bh*CFBP38 is exported by the bacterium. The domain architecture of all non-apicomplexan FCBPs and some representative CFBPs are shown in Figure S3.

**Figure 9:**
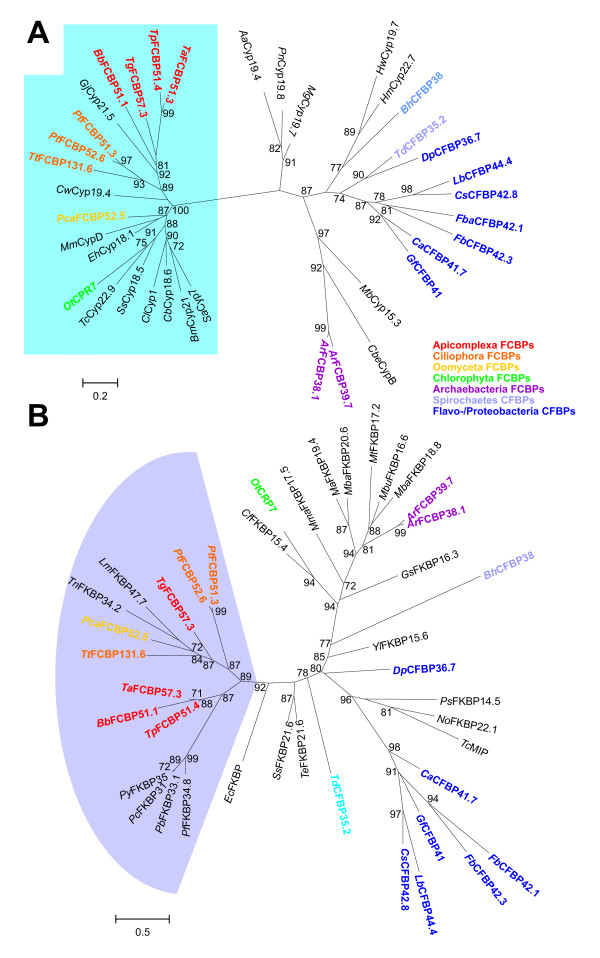
**Phylogram showing evolutionary relationships for Cyp and FKBP domains of FCBPs and CFBPs**. Cyp domains (A) and FKBP domains (B) of FCBPs and CFBPs were aligned with related domains identified by BLAST analyses in archaebacteria, eubacteria and eukaryotes. Unrooted maximum likelihood phylograms were calculated using PhyML [34]. Statistical support for branches is given as approximate likelihood ratio at the nodes. Only likelihoods of at least 70% are presented. FCBPs of apicomplexa, ciliophora, oomyceta, chlorophyta, and archaebacteria are highlighted in red, orange, yellow, green, and purple, respectively. CFBP of spirochaetes and flavo-/proteobacteria are marked in different blue tones. Species abbreviations: *Ta*, *Theileria annulata*; *Tp*, *Theileria parva*; *Tg*, *Toxoplasma gondii*; *Bb*, *Babesia bovis*; *Gj*, *Griffithsia japonica*; *Pt*, *Paramecium tetraurelia; Tt*, *Tetrahymena thermophila*; *Cw*, *Crocosphaera watsonii*; *Pca*, *Phytophora capsici*; *Mm*, *Mus musculus*; *Eh*, *Entamoeba histolytica*; *Ot*, *Ostreococcus tauri*; *Tc*, *Trypanosoma cruzi*; *Ss*, *Synechocystis spec*.; *Cl*, *Codonopsis lanceolata*; *Cb*, *Caenorhabditis briggsae*; *Bm*, *Blastupirellula marina*; *Sa*, *Stigmatella aurantiaca*; *Ar*, uncultured archaeon GZfos18C8; *Cbe*, *Clostridium beijerincki*; *Mb*, *Methanococcoides burtonii*; *Gf*, *Gramella forsetii*; *Ca*, *Croceibacter atlanticus*; *Fb*, *Flavobacteriales bacterium*; *Fba*, *Flavobacteria bacterium*; *Cs*, *Celluphaga spec*. MED134; *Lb*, *Leeuwenhoekiella blandensis*; *Dp*, *Desulfotalea psychrophilia*; *Td*, *Treponema denticulata*; *Bh*, *Borrelia hermsii*; *Hm*, *Haloarcula marismortui*; *Haloquadrantum walsbyi*; *Mg*, *Magnaporthe grisea*; *Pn*, *Phaeosphaeria nodorum*; *Aa*, *Aedes aegyptii*; *Lm*, *Leishmania major*; *Tn*, *Tetraodon nigroviridis*; *Py*, *Plasmodium yoelii*; *Pc*, *Plasmodium chabaudi*; *Pb*, *Plasmodium berghei*; *Pf*, *Plasmodium falciparum*; *Ec*, *Entodinium caudatum*; *Te*, *Trichodesmium erythraeum*; *No*, *Nitrosococcus oceani*; *Ps*, *Polaromonas spec*. Js666; *Yl*, *Yarrowia lipolytica*; *Gs*, *Geobacter spec*. FRC-32; *Mba*, *Methanosarcina barkeri*; *Mbu*, *Methanococcoides burtonii*; *Mt*, *Methanotherococcus thermolithotrophicus*; *Ma*, *Methanosarcina acetivorans*; *Mma*, *Methanoculleus marisnigri*; *Cf*, *Chlorobium ferrooxidans*.

The discontinuous distribution pattern of FCBPs and CFBPs in phylogenetically unrelated clades raises the question whether these proteins evolved multiple times independently. Alternatively, a common evolutionary origin of proteins with this domain architecture might be assumed followed by either loss from most genomes or horizontal gene transfer. In order to address this question, BLAST analyses were used to identify those Cyps and FKBPs in archaebacteria, eubacteria, and eukaryotes that show the highest similarity to the diverse FCBPs and CFBPs. All proteins used for these analyses are listed in Tables S2 and S3 in Additional file 4. Then, maximum likelihood analyses were performed independently on ClustalW2-built alignments of Cyp and FKBP domains. Results of these phylogenetic analyses are presented in Figure 9. The cyclophilin domains of all eukaryotic FCBPs are closely related (i.e. most of them are recognized as Cyp_ABH domain by CD-BLAST) and therefore form a highly significant cluster in Figure 9A (group in the dendrogram with blue background). However, they are clearly not monophyletic as there are several non-FCBP Cyps within this group and FCBP proteins have apparently evolved at least three times independently – *i.e*. in chlorophyta, oomycetes and alveolata. For *Ot*CPR7 this conclusion is further supported by the fact that this FCBP does not contain any TRP repeats. Cyp domains of the putative archaebacterial FCBPs are not even closely related to this group and form a completely independent cluster. The Cyp domains of proteo-/flavobacterial CFBP proteins are monophyletic – in contrast to those of spirochaetes. However, for the latter group there are currently only members known from *Treponema denticula *and four *Borrelia *species. It is for instance possible that one of these two proteins is highly divergent from the average spirochaete CFBP due to secondary evolutionary changes. In particular, the presence of a lipoprotein anchor at the NH_2_-terminus of *Bh*CFBP38 suggests an extracellular localization of the mature protein and therefore a significantly altered function.

The Cyp domains of FCBPs of ciliophora and apicomplexa are closely related, surprisingly, however, a non-FKBP Cyp from the rhodophyte *Griffithsia japonica *is proposed to be a member of the same cluster as revealed by maximum likelihood analysis. Since red algae are frequently supposed to be the evolutionary origin of the apicoplast, one explanation for this result may be that the Cyp domain of FKBPs in alveolata was derived from the genome of a rhodophyte-related secondary endosymbiont.

Phylogenetic analysis of the deduced FCBP domains does also not support a monophylic origin of alveolate FCBPs. Overall, phylogenetic distances between FKBP domains are much larger than for Cyp domains (compare scale bar between Figures 9A and 9B) indicating that the latter are far better conserved. Moreover, the phylogram reveals poor sequence conservation even within groups containing a well conserved Cyp domain. For instance, the CFBPs of proteo-/flavobacteria do not form a monophyletic group when FKBP domains are analyzed (Figure 9B). The two spirochaete CFBPs are clearly separated and the green algal *Ot*CPR7 does not show any close relationship with FKBP domains from other eukaryotic FCBPs but appears to be closely related to bacterial FKBPs suggesting that it might have been acquired from a cyanobacterial endosymbiont. Though the FKBP domains of all alveolat FCBPs can be found in the same highly significant cluster (group with blue background), this group also contains non-FCBP FKBPs. Conspicuously, however, all FKBP proteins within this group also contain TRP repeats (compare Figures 8, S3, 9B, and Table S3). In contrast to the results obtained for Cyp domains, not even the FKBP domains of FCBPs from ciliophora and apicomplexa appear to be monophyletic. On one hand, it is quite unlikely that the same structure of FKBP domain and Cyp domain connected by TRP repeats arose multiple times independently and it can therefore be suspected that this result is due to high and diversifying evolutionary pressure on FKBP domains in this protein family. On the other hand, there is a widely distributed monophyletic family of FKBP proteins with TRP repeats. These proteins might indeed have captured a Cyp domain several times independently. The strongest argument for the latter evolutionary pathway is the presence of putative FKBP proteins with TRP repeats and a concurrent absence of predicted FCBP proteins in all *Plasmodium *species. If FCBP proteins would be a monophyletic, ancient group in alveolata or at least in apicomplexa, secondary loss of the Cyp domain from FCBPs restoring the ancient FKBP/TRP protein has to be postulated for *Plasmodium*.

In summary, additional data from more species are needed to draw a conclusive pattern of evolution for FCBP proteins in protists. Currently, there are hints supporting either a monophyletic origin or multiple independent origins though the latter option appears to be slightly more likely.

### Cyps with WD40 repeats

**Figure 10:**
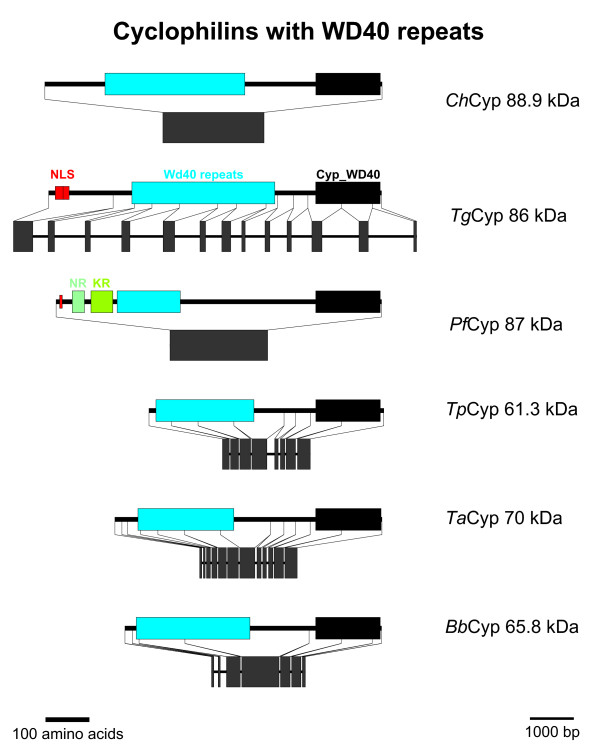
**Cyps with WD40 repeats**. Domain architecture and genomic organization of Cyps with WD40 repeats. Species are abbreviated as in Fig. 1. NLS, nuclear localization signal; WD40 repeat (CD accession-no.: [cl02567]), Cyp_ABH, ABH-type Cyp domain (CD accession-no.: [cd01926]); NR, Asp-rich region; KR, Lys-rich region.

The first group of putative Cyps with a non-Cyp_ABH domain is represented by the WD40 repeat-containing Cyps which are encoded in all apicomplexan genomes analyzed here (Figure 1). This subfamily contains multi-domain Cyps with a Cyp_WD40 domain (CD accession-no: [cd01927]) in its COOH-terminus (Figure 10). WD40 domains (CD accession no.: [cl02567]) are characterized by repeats of about 40 amino acids containing a characteristic Trp/Asp dipeptide. The length of the deduced WD40 domain ranges from 145 amino acids in *P. falciparum *to 321 and 328 amino acids in *C. hominis *and *T. gondii*, respectively. The WD40 repeat domain is either located close to the NH_2_-terminus as in *Theileria *species and in *B. bovis*, or is preceded by a longer NH_2_-terminal extension without identifiable domains or motifs as in *T. gondii*, *P. falciparum*, and *C. hominis*. There is no sequence similarity between the orthologs within this region. In *T. gondii*, however, a nuclear localization signal can be found here, and in *P. falciparum *there are two short stretches rich in Asn and Lys, respectively. An orthologous protein from *E. tenella *has recently been described to contain a very Ser- and His-rich NH_2_-terminus [29].

The genomic organization differs largely between species with intronless genes in *C. hominis *and *P. falciparum *while there are between 6 (*B. bovis*) and 12 introns (*T. gondii*) in the other species.

Deckert *et al*. [53] showed that the human WD40-repeat Cyp is a component of the spliceosomal B complex which contains the complete set of U snRNAs in a precatalytic state. However, its precise role in splicing or regulation of splicing has not been addressed yet. Recent structural analyses show that – in the crystal – the NH_2_-terminus of the protein binds to the active site of a neighboring molecule in a substrate-analogous manner [54]. Binding of this sequence to the active center without subsequent isomerization was also confirmed by NMR solution studies.

### PPIL1-like Cyps

For Cyps of the PPIL1/*Sp*Cyp2 subfamily, CD-BLAST does not recognize any special Cyp domain but only the Cyp superfamily in general (accession-no.: [cl00197]). Only two putative members of this subfamily can be found in the apicomplexan genomes analyzed here, i.e. *Tg*Cyp21 and *Pf*Cyp23.2 (Figure 1 and Figure S4 in Additional file 6). *Pf*Cyp23.2 is also predicted to have an NH_2_-terminal coiled-coil region. However, there is no hint for such a domain in other PPIL1-like Cyps such as *Tg*Cyp21 or *Sp*Cyp2.

*Hs*PPIL1 has been demonstrated to be a part of the spliceosomal machinery [38] and to directly interact with the highly conserved transcriptional cofactor SKIP [55]. Although PPIL1-like Cyps are widely spread, they are for instance missing in a large number of fungal genomes [9] indicating that the function of this subfamily is not essential. Since SKIP is also involved in splicing and remains bound to the spliceosome throughout both trans-esterification steps [55], PPIL1-like Cyps and SKIP might be involved in the complex linkage of transcription and splicing during mRNA processing.

### PPIL3-like Cyps

**Figure 11:**
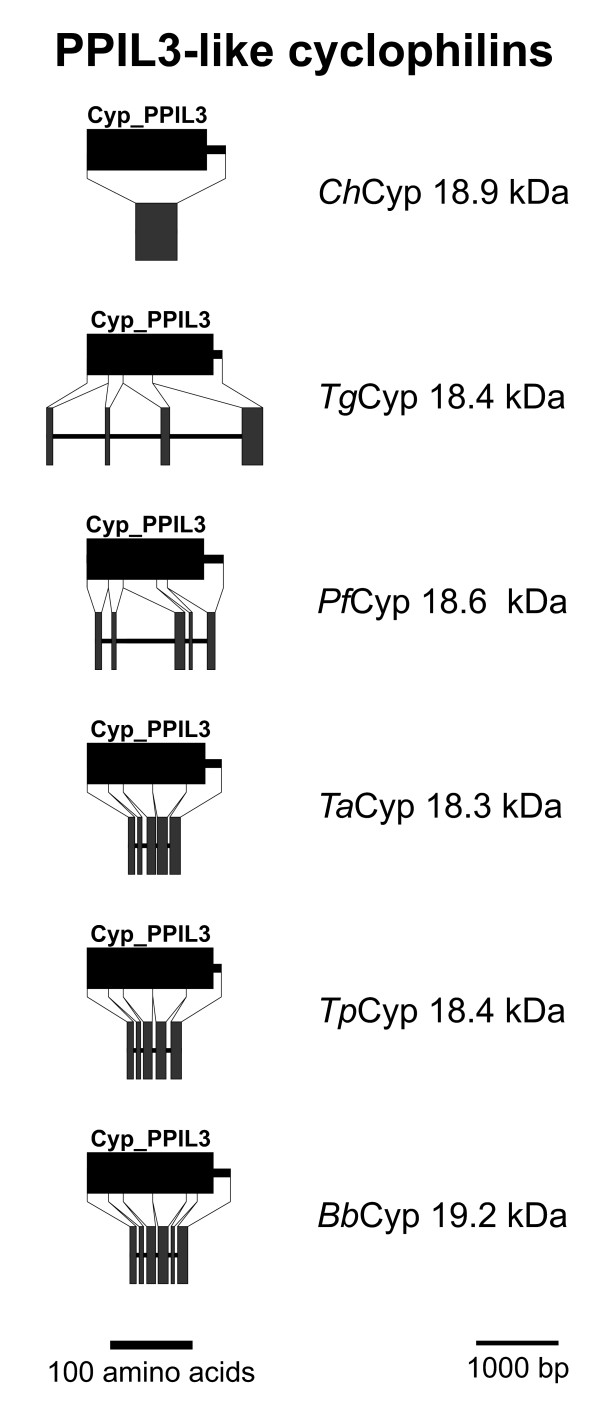
**PPIL3-like Cyps**. Domain architecture and genomic organization of PPIL3-like Cyps. Species are abbreviated as in Fig. 1. Cyp_PPIL3, PPIL3-type Cyp domain (CD accession-no: [cd01928]).

Within the non Cyp_ABH group, the PPIL3-like Cyps are the only subfamily of small single domain Cyps that is widely distributed among apicomplexa. PPIL3-like Cyps can be predicted in all the apicomplexan genomes and consist of little more than the Cyp_PPIL3 domain (accession-no.: [cd01928]) itself (Figures 1 and 11). Since apparent localization signals are missing, a predominant cytosolic localization of the putative proteins might be assumed. However, at least one splice form of human PPIL3 has been identified as part of the B complex of the spliceosome in the nucleus [38] though its precise role in the spliceosome B complex has not been analyzed yet.

In contrast to the PPIA-like subfamily, the major subfamily of small cytosolic Cyps, most of the PPIL3-like Cyps are encoded by genes consisting of four (*P. falciparum*) to six (*B. bovis*) exons (Figure 11). In this case, the only exception is *Ch*Cyp18.9, which has an intronless coding sequence.

### PPIL2-like Cyps

**Figure 12:**
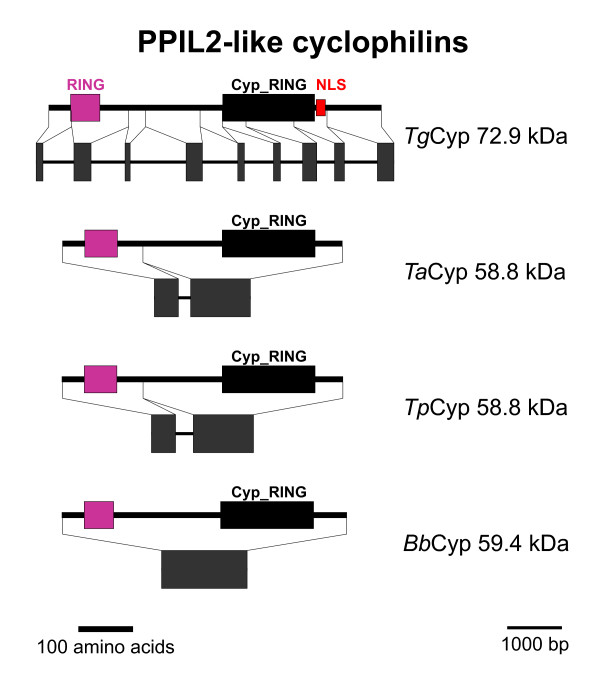
**PPIL2-like Cyps**. Domain architecture and genomic organization of Cyps with RING finger domain. Species are abbreviated as in Fig. 1. RING, RING finger domain (Interpro accession-no.: IPR003613); Cyp_RING, RING-type Cyp domain (CD accession-no: [cl00197]); NLS, nuclear localization signal.

Putative PPIL2-like Cyps form a very robust phylogenetic cluster (Figure 1) and are characterized by the presence of a so-called RING finger domain that has been reported to facilitate E3 ubiquitin-ligase activity [56]. Cyps with RING finger motif in their NH_2_-terminus are widely spread among different organisms including fungi, plants and mammals, and the *Arabidopsis thaliana *ortholog, *At*PUB49, has been shown to be an active E3 ubiquitin-ligase and to exhibit PPIase and chaperone activity suggesting that it is involved in protein folding and degradation processes. The Cyp_RING domain (accession-no: [cd01923]) is present in the vicinity of the COOH-terminus (Figure 12). This subfamily is apparently missing in the genomes of *P. falciparum *and *C. hominis*. The deduced sequence of *Tg*Cyp72.9 is somewhat larger than its orthologs in *Theileria *and *Babesia *(between 58.8 and 59.4 kDa) due to a longer COOH-terminus. Moreover, *Tg*Cyp72.9 contains a putative nuclear localization signal which is not present in its haemosporidian orthologs.

The genomic organization of Cyps with RING finger domain again shows signs of intron-loss during evolution with eight introns in *T. gondii*, one intron in both *Theileria *species and no intron left in *B. bovis*.

### CeCyp16-like Cyps

**Figure 13:**
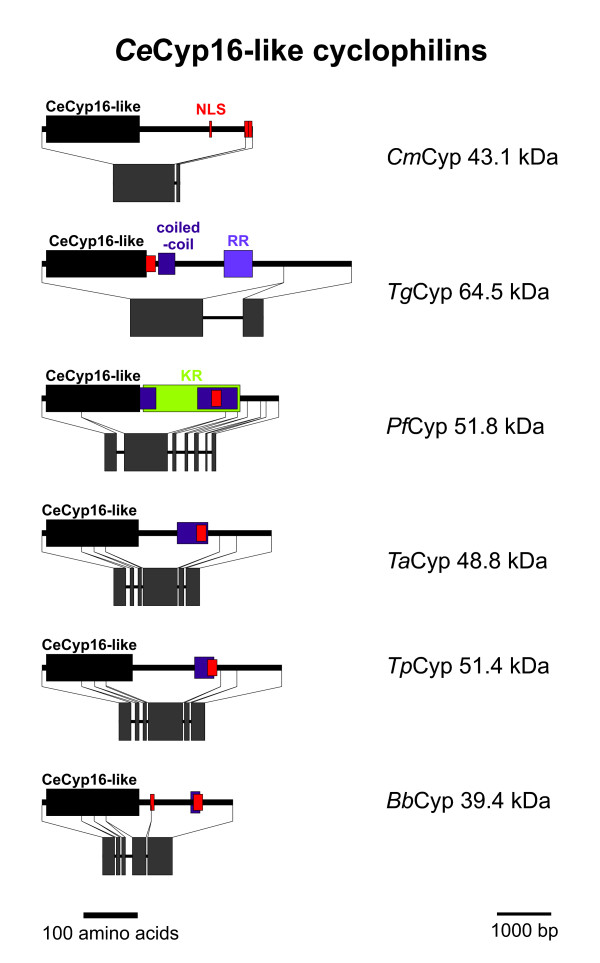
***Ce*Cyp16-like Cyps**. Domain architecture and genomic organization of *Ce*Cyp16-like Cyps. Species are abbreviated as in Fig. 1. Cyp_*Ce*Cyp16, *Ce*Cyp16-type Cyp domain (CD accession-no: [cd01925]); NLS, nuclear localization signal; coiled-coil, coiled-coil protein interaction region; RR, Arg-rich region; KR, Lys-rich region.

In contrast to most other moderate to large size Cyps, the subfamily containing a Cyp-*Ce*Cyp16-like domain (accession number: [cd01925]) does not contain any additional domain that could be identified by CD-BLAST or InterProScan (Figure 13). However, there is a nuclear localization signal detectable in all putative apicomplexan *Ce*Cyp16-like Cyps, which is located in approximately the same distance from the Cyp domain in all subfamily members with the exception of *Tg*Cyp64.5 where it immediately follows the Cyp domain. Moreover, using PSORTII at least one coiled-coil protein-protein interaction domain can be identified in all these proteins but *Cm*Cyp43.1 (for *Cm*Cyp43.1 the score for a coiled-coil region is only slightly too small to be judged as significant by PSORTII). *Pf*Cyp51.8 is even predicted to contain two coiled-coil regions. Moreover, *Pf*Cyp51.8 has a large Lys-rich region encompassing both coiled-coil domains. A comparable but much smaller positively charged region consisting of a large number of Arg residues is present in *Tg*Cyp64.5.

The *C. elegans *ortholog *Ce*Cyp16 has been shown to be expressed predominantly in the intestine [57] and high-troughput RNAi screening experiments revealed strong phenotyps for *Ce*Cyp16 including lethality, embryonic lethality, morphological abnormalities and maternal sterility [58,59] indicating that this subfamily of Cyps is very important at least in multi-cellular organisms. However, since *Ce*Cyp16 is much smaller than its apicomplexan orthologs and most of the latter miss a clearly definable positively charged domain in their COOH-terminus that can be found in nematode *Ce*Cyp16-like proteins [57], it is not yet possible to draw any conclusions regarding the function of *Ce*Cyp16-like Cyps in apicomplexa. Even certain biochemical observations made on recombinant *Ce*Cyp16-like proteins from the nematodes *C. elegans *and *Onchocerca volvolus *cannot simply be extended to their ortologs in apicomplexa. In particular, although both nematode and apicomplaxan *Ce*Cyp16-like Cyps reveal absence of an otherwise highly conserved Trp residue in the active center of the enzyme, there are acidic residues in this position in nematode *Ce*Cyp16-like proteins but a wide variety of different amino acids residues in *Ce*Cyp16-like Cyps of apicomplexa including Gln, Val, Tyr, Cys, and Phe. Since the conserved Trp residue has been shown to be crucial for CsA binding/sensitivity, it can therefore be assumed that *Ce*Cyp16-like proteins of apicomplexa are in general rather resistant to CsA. However, predictions about changes in substrate specificity cannot be made using the results of biochemical analyses made on *C. elegans *and *O. volvolus *orthologs.

### PPIL4-like Cyps

Putative PPIL4-like Cyps are only identifiable in the genomes of *C. hominis *and *T. gondii *(Figures 1 and Figure S5 in Additional file 7). Although orthologs are present in other *Cryptosporidium *species (data not shown) and many but not all genomes of fungi [9], this subfamily is apparently absent from all other apicomplexan genomes analyzed. *Ch*Cyp34.5, just like its orthologs *Sp*Cyp6 and *Hs*PPIL4, contains an RRM (RNA recognition motif) closer to its COOH-terminus and a Cyp_RRM domain (accession-no.: [cd01921]) in its immediate NH_2_-terminus (Figure S5). In contrast to other PPIL4-like Cyps, the deduced sequence of *Ch*Cyp34.5 is missing a Ser/Arg-rich SR domain in its COOH-terminus. The predicted sequence of *Tg*Cyp66.3 is very unusual since it contains a Cyp domain that is interrupted by a large insertion which remains to be confirmed experimentally. However, in contrast to *Ch*Cyp34.5, *Tg*Cyp66.2 is more typical for PPIL2-like Cyps since it possesses a Ser-rich and highly positively charged domain in its COOH-terminus. Although it also contains multiple Lys in addition to Arg residues, it can be assumed that this domain fulfills a function similar to that of the SR domain of mammalian and fungal PPIL4-like Cyps. It is not unlikely that both *Ch*Cyp34.5 and *Tg*Cyp66.3 are not yet predicted accurately (e.g. a missing exon in *Ch*Cyp34.5 and a missing intron in *Tg*Cyp66.3 would explain the current results) and it will finally turn out that both possess normal Cyp domains and an SR domain.

PPIL4-like Cyps should not be confused with the PPIE-like Cyps, a subfamily that is missing in all apicomplexan genomes. PPIE-like Cyps contain an RRM motif in the NH_2_-terminus and a Cyp_ABH domain in their COOH-terminus.

*Ch*Cyp34.5 contains a nuclear localization signal within its Cyp domain and PSORTII predicts a nuclear localization. Due to its high content of positively charged amino acid residues, the putative *Tg*Cyp66.3 is predicted to have a multitude of overlapping nuclear localization signals in its COOH terminus in addition to one signal about 100 amino acids away from its NH_2_-terminus. Indeed, the orthologous *At*Cyp59 protein from *A. thaliana *has been described to be localized in the nucleus but outside of those nuclear speckles rich in SR domain proteins [60]. Although interaction with other SR domain proteins implicated in RNA splicing could be demonstrated using yeast-two-hybrid and pull-down assays, the punctuate nuclear localization pattern and a measurable interaction with the COOH-terminal domain of RNA polymerase II suggest that *At*Cyp59 predominantly participates in transcriptional processes and that it is only marginally involved in splicing [60]. It is still too early to speculate whether PPIL2-like Cyps of apicomplexa have similar functions as *At*Cyp59 or other PPIL2-like Cyps since the SR domain responsible for all known *At*Cyp59 interactions is missing in *Ch*Cyp34.5 and the Cyp domain of *Tg*Cyp66.3 might well be non-functional. In this context it is also noteworthy that the Cyp domain of this protein is less conserved than that of other PPIL4-like Cyps. Whereas this domain in *Sp*Cyp6 and *Hs*PPIL4 is recognized as Cyp_RRM domain (accession no.: [cd01921]), CD-Blast only recognizes a domain belonging to the Cyp superfamily (accession no.: [cd00197]). It is also possible that the truncated or disrupted PPIL4-like Cyps in *Cryptosporidia *and *Toxoplasma *containing a slightly degenerated Cyp domain represent transition states that ultimately led to complete loss of this gene in other apicomplexa.

### Genomic organization of Cyp genes

Loss of introns during evolution of organisms exhibiting a parasitic live mode such as Giardia [61], Trypanosoma [62], Trichomonas [63], and Encephalitozoon [64] has been recognized previously and has also been described for apicomplexan parasites on a genome wide scale [65]. While Toxoplasma is known to have a genomic organization with a very high number of about five introns on average per gene, Theileria and Plasmodium species have approximately two and one intron on average per gene. Only a very small number of introns can be found in the genomes of Cryptosporidium species which have introns in only about 5% of their genes. However, most of the few remaining introns in Cryptosporidium genomes are not conserved to T. gondii introns and are therefore supposed to be not of ancient origin [65]. Apparently, loss of introns occurred independently in the lineages leading to crypto- and haemosporidia.

As expectable from the differences in the general genomic organization, the exon/intron structure of Cyp genes varies widely between species. The number of introns per Cyp gene, i.e. 0.5 for *C. hominis*, 4.5 for *T. gondii*, 2.0 for *P. falciparum*, 3.1 for *T. parva*, 3.5 for *T. annulata*, and 2.8 for *B. bovis *is very similar to that observed on the genome scale. Despite this overall similarity, however, there are some remarkable exeptions from the general pattern: First, the putative *Ch*Cyp22.9 with SP in *C. hominis *shows a conserved position of introns in comparison to its ortholog in *T. gondii*. Secondly, some Cyp subfamilies have a high number of introns in both haemosporidians and *T. gondii *although only minimal conservation of introns can be observed. This group includes the small apicomplexa-specific cyclophilins, the Cyps with WD40 repeats (with exception of *Pf*Cyp87), and the PPIL3-like Cyps. Finally, there is one Cyp gene subfamily, the *Ce*Cyp16-like Cyps, showing a completely reversed trend with more introns in the haemosporidian genomes than in *T. gondii*. Since several of the introns in haemosporidia appear to be of ancient origin, it is most likely that *Tg*Cyp65.5 has lost its introns since divergence from the last common anchestor with the haemosporidia.

In summary, though the trend of moderate and nearly complete loss of introns observed on a genome-wide scale for haemo- and cryptosporidia, respectively, could also be observed for Cyp genes in general, there are exceptions to this rule in certain subfamilies that might be exploited in the future to decipher the selection forces that contribute to conservation of certain introns despite high overall frequency of intron loss. For instance, it would be highly intriguing to look for any functional roles for the three introns in *Ch*Cyp22.9 (e.g. on regulation of gene expression) that might explain counterselection against their deletion during evolution.

## Conclusion

The present study was able to identify 16 different Cyp subfamilies in apicomplexa. While some of these subfamilies can be found in the genomes of all species analyzed, there are also two small subfamilies, that can only be found in the genus *Cryptosporidium *and *Toxoplasma *(PPIL4-like Cyps) or even only in *Toxoplasma *(PPIL6-like Cyps), respectively. Six out of these 16 subfamilies (*i.e*. PPIH-like, SYF2-containing, WD40-containing, PPIL-3-like, PPIL-4-like, and PPIL-1-like Cyps) have been described to be a part of the extremely complex transcription and/or splicing machinery in mammalian or yeast cells indicating that regulation of protein conformation in these very large protein or ribonucleoprotein complexes catalyzing RNA processing is a highly conserved major function of eukaryotic Cyps.

While most apicomplexa are predicted to posses typical cytoplasmic PPIA-like Cyps, these putative proteins in both *Theileria *species are predicted to have an NH_2_-terminal apicoplast localization signal. Surprisingly, these are the only Cyps that are predicted to be transported to the apicoplast. Apicomplexa might be more easily able to cope with loss of cytosolic PPIA-like proteins than other eukaryota since members of the apicomplexa-specific group of relatively small Cyps with Cyp_ABH domain might be able to functionally replace PPIA-like cytosolic Cyps. Moreover, at least one member of the Cyp subfamily with signal peptides has been reported not to be confined to the secretory pathway but to be present in the cytosol as well [24]. This Cyp subfamily is very closely related to cytosolic PPIA-like Cyps and therefore unique in so far as it does not represent orthologs of the PPIB-like subfamily that is present in the secretory pathway of other eukaryotes.

Since the Cyp antagonist CsA has been shown to have anti-parasitc activity against a wide variety of apicomplexa [13,16,17,19,20], Cyps represent an attractive target for the identification of new drugs against this important group of pathogens. These might either include non-immunosuppressive CsA derivatives or completely new, structurally unrelated agents. Systematic identification and characterization of the apicomplexan Cyp repertoire as commenced in this bioinformatic survey will enable future analysis of suitable drug targets in more detail. The encouraging fact that there are Cyp subfamilies that are absent from their mammalian hosts, such as Cyps with signal peptides, small apicomplexa-specific Cyps, *Plasmodium*-specific Cyps, and Cyps with SYF2 domain, already points out obvious drug target candidates.

## Methods

### Identification of Cyp genes

Initially, putative apicomplexan Cyps were identified using BLASTp and tBLASTn algorithms to search in GenBank^® ^protein and nucleic acid databases as well as in PlasmoDB, ToxoDB, CryptoDB, and in the *Theileria parva *genome database of TIGR. *S. pombe *Cyp1 and Cyp2 were used as query sequences. These Cyps were chosen because they are not closely related. If a Cyp subfamily member was not identified in one of the apicomplexan organisms, a Cyp of the same subfamily from a closely related apicomplexan parasite was used as query to search in protein, cDNA, EST and genome databases. This method ensures that no Cyps are missed in any of the taxa. In order to prevent that no complete subfamilies was overseen, BLAST analyses were also performed using the complete *T. gondii *Cyp repertoire as a query. However, no additional Cyp sequences could be identified.

In contrast to conventional nomenclature for many Cyps, molecular mass suffixes in the names were given with one position after the decimal point since otherwise identical names would have resulted in a few cases. It was decided not to use suffix letters to avoid a possible confusion with mammalian Cyps. For instance, a Cyp19A might have been confused with a human CypA/PPIA. In addition it should be mentioned that all molecular mass suffixes used have been derived from the predicted sequence of unprocessed proteins. Although this can currently be only a provisional nomenclature, consecutive naming with numbers or letters would result in different names for orthologues Cyps and identical names for unrelated Cyps of different apicomplexa. A more function based nomenclature of apicomplexan Cyps should be introduced later, when at least for one apicomplexan genome all Cyps have been verified experimentally. For human and *S. pombe *Cyps, names according to the entries in the ENSEMBL database were used.

### Phylogenetic analyses

Homologous putative protein sequences were aligned using ClustalW2 [33]. Maximum likelihood phylogenetic trees were then calculated with PhyML [34] using the approximate likelihood ratio test option and the JTT model [66] for amino acid substitution. The program was set to estimate the proportion of invariable sites and the gamma distribution parameter, while the number of substitution rate categories was set to four. The input tree was built using the BIONJ algorithm implemented in PhyML. The resulting trees in Newick format were visualized and processed using MEGA4 [67,68].

### Identification of protein domains

For identification of protein domains, CD-BLAST [31,32] and InterPro Scan [69] were used. Moreover, protein sequences were scanned for subcellular localization signals with PSORT, SignalP [70], PATS [40], PlasMit [45,71], and Mitoprot [44].

## Abbreviations

ABC: ATP-binding casette; AP: apicoplast transit signal; CFBP: cyclosporin A- and FK506-binding proteins; CsA: cyclosporin A; Cyp: cyclophilin; ER: endoplasmic reticulum; FCBP: FK506- and cyclosporin A-binding proteins; FK506: tacrolimus; FKBP: FK506-binding proteins; ORF: open reading frames; PPIase: peptidyl-prolyl *cis/trans *isomerase; snRNP: small nuclear ribonucleoprotein particle; SP: signal peptide; SR domain: Ser- and Arg-rich domain.

## Competing interests

The authors declare that they have no competing interests.

## Authors' contributions

JK, GG, and GvSH designed the study and wrote the final manuscript. JK performed bioinformatic analyses and drafted the manuscript. All authors approved the final version.

## Supplementary Material

Additional file 1 **Table S1: Cyclophilins from *H. sapiens *and *S. pombe***. Listing of the human and fission yeast Cyp repertoire used for comparison with apicomplexan Cyps. Accession-no., protein size and domain architecture are summarized. Click here for file

Additional file 2 **Figure S1 – *Plasmodium*-specific Cyps**. Domain architecture and genomic organization of *Plasmodium*-specific Cyps. Click here for file

Additional file 3 **Figure S2 – PPIL6-like Cyp *Tg*Cyp36.7**. Domain architecture and genomic organization of *Tg*Cyp36.7. Click here for file

Additional file 4 **Tables S2 and S3: Protein sequences used to analyse evolution of Cyp and FKBP domains in dual class immunophilins**. Listing of the genes used for comparison with apicomplaxan FKBPs in Figures 5 and 6. Click here for file

Additional file 5 **Figure S3 – FCBP and CFBP proteins in non-apicomplexa**. Domain architecture of FCBPs and CFBPs from non-apicomplexan organisms. Click here for file

Additional file 6 **Figure S4 – PPIL1-like Cyps**. Domain architecture and genomic organization of PPIL1-like Cyps. Click here for file

Additional file 7 **Figure S5 – PPIL4-like Cyps**. Domain architecture and genomic organization of *Ch*Cyp34.5, the only apicomplexan PPIL4-like Cyp which contains an RNA recognition motif. Click here for file
