# Supplementary material for: In silico analysis of the cyclophilin repertoire of apicomplexan parasites
Source: Parasit Vectors. 2009 Jun 25;2:27. doi: 10.1186/1756-3305-2-27 (PMC2713222; doi:10.1186/1756-3305-2-27)
Supplement: Additional file 1 — Table S1: Cyclophilins from H. sapiens and S. pombe. Listing of the human and fission yeast Cyp repertoire used for comparison with apicomplexan Cyps. Accession-no., protein size and domain architecture are summarized. [file 1756-3305-2-27-S1.pdf]

Tab. S1: Cyclophilins from *H. sapiens* and *S. pombe*

Name	Entrez Gene Accession -no.	Species	Group	Size (amino acids/kDa)	Domains[a]
*Hs*PPIA1	5478	*Homo sapiens*	Vertebrata	165/18.0	Cyp
*Hs*PPIAL4A	164022	*Homo sapiens*	Vertebrata	164/18.2	Cyp
*Hs*PPIAL4B	653505	*Homo sapiens*	Vertebrata	164/18.2	Cyp
*Hs*PPIAL4C	653598	*Homo sapiens*	Vertebrata	164/18.2	Cyp
*Hs*PPIAL4E	730262	*Homo sapiens*	Vertebrata	164/18.2	Cyp
*Hs*PPIAL4F	728945	*Homo sapiens*	Vertebrata	164/18.2	Cyp
*Hs*PPIAL4G	644591	*Homo sapiens*	Vertebrata	164/18.2	Cyp
*Hs*PPIB	5479	*Homo sapiens*	Vertebrata	216/23.7	Cyp
*Hs*PPIC	5480	*Homo sapiens*	Vertebrata	212/22.8	Cyp
*Hs*PPID	5481	*Homo sapiens*	Vertebrata	370/40.8	Cyp<br>TRP
*Hs*PPIE	10450	*Homo sapiens*	Vertebrata	301/33.4	RRM<br>Cyp
*Hs*PPIF	10105	*Homo sapiens*	Vertebrata	207/22.0	Cyp
*Hs*PPIG	9360	*Homo sapiens*	Vertebrata	247/27.5	Cyp
*Hs*PPIH	10465	*Homo sapiens*	Vertebrata	177/19.3	Cyp
*Hs*PPIL1	51645	*Homo sapiens*	Vertebrata	166/18.2	Cyp
*Hs*PPIL2	23759	*Homo sapiens*	Vertebrata	520/58.8	RING<br>Cyp
*Hs*PPIL3	53938	*Homo sapiens*	Vertebrata	161/18.1	Cyp
*Hs*PPIL4	85313	*Homo sapiens*	Vertebrata	492/57.2	Cyp<br>RRM
*Hs*PPIL6	285755	*Homo sapiens*	Vertebrata	311/35.2	
*Hs*PPWD1	23398	*Homo sapiens*	Vertebrata	646/73.6	WD40<br>Cyp
*Hs*SDCCAG10	10283	*Homo sapiens*	Vertebrata	472/53.8	Cyp
*Hs*NKTR	4820	*Homo sapiens*	Vertebrata	1462/165.7	Cyp
*Sp*Cyp1	2543019	*Schizosaccharomyces pombe*	Fungi	155/16.7	Cyp
*Sp*Cyp2	2540269	*Schizosaccharomyces pombe*	Fungi	162/17.4	Cyp
*Sp*Cyp3	2540198	*Schizosaccharomyces pombe*	Fungi	173/18.9	Cyp
*Sp*Cyp4	2541395	*Schizosaccharomyces pombe*	Fungi	201/22.2	Cyp
*Sp*Cyp5	2542541	*Schizosaccharomyces pombe*	Fungi	356/40.2	Cyp<br>TPR<br>NLS
*Sp*Cyp6	2539942	*Schizosaccharomyces pombe*	Fungi	432/50.8	Cyp<br>RRM
*Sp*Cyp7	2540067	*Schizosaccharomyces pombe*	Fungi	463/52.2	Cyp
*Sp*Cyp8	2541969	*Schizosaccharomyces pombe*	Fungi	471/53.6	RING<br>Cyp
*Sp*Cyp9	2539557	*Schizosaccharomyces pombe*	Fungi	610/69	WD40<br>Cyp

[a]Cyp, cyclophiln domain; RRM, RNA recognition motif; RING, RING finger domain; TRP, Tetratricopeptide repeat; WD40, WD40 repeat.
