# Supplementary material for: In silico analysis of the cyclophilin repertoire of apicomplexan parasites
Source: Parasit Vectors. 2009 Jun 25;2:27. doi: 10.1186/1756-3305-2-27 (PMC2713222; doi:10.1186/1756-3305-2-27)
Supplement: Additional file 2 — Figure S1 – Plasmodium-specific Cyps. Domain architecture and genomic organization of Plasmodium-specific Cyps. [file 1756-3305-2-27-S2.pdf]

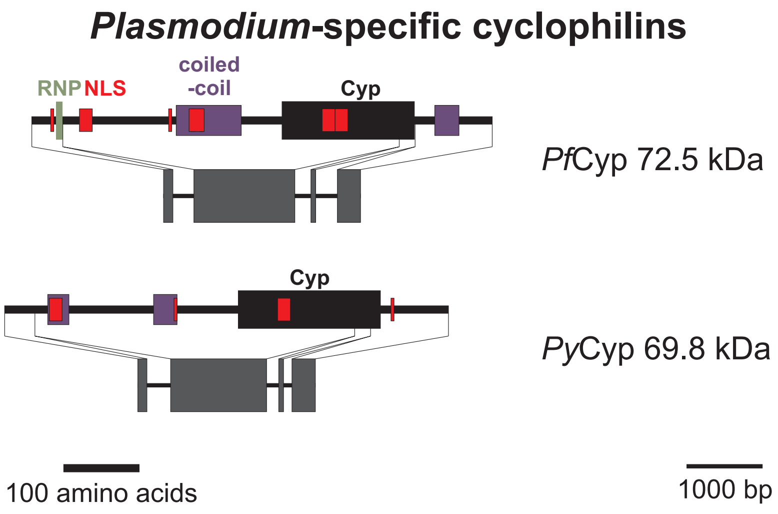

Figure S1 - *Plasmodium*-specific Cyps

Domain architecture and genomic organization of *Plasmodium*-specific Cyps. Species are abbreviated as in Fig. 1. Cyp, Cyp domain (CD accession-no.: [cd00317]); RNP, RNA-binding motif (InterPro accession-no.: [IPR000504]) coiled-coil, coiled-coil protein interaction domain; NLS, nuclear localization signal.
