# Supplementary material for: In silico analysis of the cyclophilin repertoire of apicomplexan parasites
Source: Parasit Vectors. 2009 Jun 25;2:27. doi: 10.1186/1756-3305-2-27 (PMC2713222; doi:10.1186/1756-3305-2-27)
Supplement: Additional file 3 — Figure S2 – PPIL6-like Cyp TgCyp36.7. Domain architecture and genomic organization of TgCyp36.7. [file 1756-3305-2-27-S3.pdf]

PPIL6-like *Tg*Cyp36.7

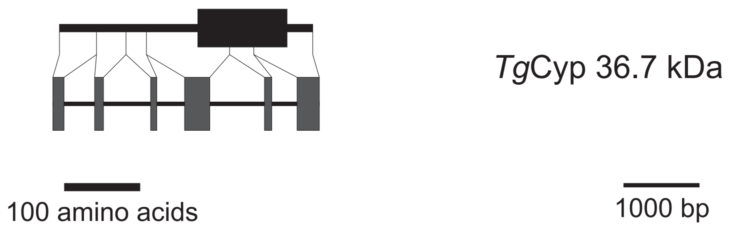

Figure S2 - PPIL6-like Cyp *Tg*Cyp36.7

Domain architecture and genomic organization of *Tg*Cyp36.7. Species are abbreviated as in Fig. 1. Cyp, Cyp domain (CD accession-no.: [cd00137]).
