# Supplementary material for: In silico analysis of the cyclophilin repertoire of apicomplexan parasites
Source: Parasit Vectors. 2009 Jun 25;2:27. doi: 10.1186/1756-3305-2-27 (PMC2713222; doi:10.1186/1756-3305-2-27)
Supplement: Additional file 4 — Tables S2 and S3: Protein sequences used to analyse evolution of Cyp and FKBP domains in dual class immunophilins. Listing of the genes used for comparison with apicomplaxan FKBPs in Figures 5 and 6. [file 1756-3305-2-27-S4.pdf]

Table S2: Protein sequences used to analyse evolution of cyclophilin domains in dual class immunophilins

Name	Accession-no.	Species	Group	Size (amino acids/kDa)	Domains[a]
*Ta*FCBP51.3	XP_951815	*Theileria annulata*	Apicomplexa	459/51.3[b]	FKBP<br>TPR repeats<br>cyclophilin
*Tp*FCBP51.4	XP_765469	*Theileria parva*	Apicomplexa	460/51.4	FKBP<br>TPR repeats<br>cyclophilin
*Tg*FCBP57.3	AAX51680	*Toxoplasma gondii*	Apicomplexa	521/57.3	FKBP<br>TPR repeats<br>cyclophilin
*Bb*FCBP51.1	YP_001883841	*Babesia bovis*	Apicomplexa	339/51.1	FKBP<br>TPR repeats<br>cyclophilin
*Gj*Cyp21.5	AAM93985	*Griffithsia japonica*	Rhodophyta	198/21.5	Single cyclophilin
*Pt*FCBP51.3	XP_001444554	*Paramecium tetraurelia* strain d4-2	Ciliophora	456/51.3	FKBP<br>TPR repeats<br>cyclophilin
*Pt*FCBP52.6	XP_001445268	*Paramecium tetraurelia* strain d4-2	Ciliophora	467/52.6	FKBP<br>TPR repeats<br>cyclophilin
*Tt*FCBP131.6	XP_976669	*Tetrahymena thermophila*	Ciliophora	1134/131.6	ATB-binding<br>FKBP<br>TRP<br>cyclophilin
*Cw*Cyp19.4	ZP_00517449	*Crocosphaera watsonii*	Cyanobacteria	174/19.4	Single cyclophilin
*Pca*FCBP52.5	BT031867	*Phytophora capsici*	Oomycetes	479/52.5	FKBP<br>TPR repeats<br>cyclophilin
*Mm*CypD	NP_080628	*Mus musculus*	Vertebrata	370/40.7	Cyclophlin<br>TRP repeats
*Eh*Cyp18.1	EAL51109	*Entamoeba histolytica*	Entamoebidae	167/18.1	Single cyclophilin
*Ot*CPR7	CAL55134	*Ostreococcus tauri*	Chlorophyta	>266 (only partial sequence)	FCBP<br>cyclophilin
*Tc*Cyp22.9	XP_808570	*Trypanosoma cruzi*	Kinetoplastida	210/22.9	Single cyclophilin
*Ss*Cyp18.5	NP_441161	*Synechocystis sp.* PCC 6803	Cyanobacteria	171/18.5	Single cyclophilin
*Cl*Cyp1	AAV48823	*Codonopsis lanceolata*	Spermatophyta	174/18.9	Single cyclophilin
*Cb*Cyp18.6	39582840	*Caenorhabditis briggsae*	Nematoda	172/18.6	Single cyclophilin
*Bm*Cyp21	ZP_01088483	*Blastopirellula marina*	Bacteria, Planctomycetes	199/21.0	Single cyclophilin
*Sa*Cyp7	ZP_01467010	*Stigmatella aurantiaca*	Bacteria, Proteobacteria	171/18.8	Single cyclophilin
*Ar*FCBP38.1	AAU82569	uncultured archaeon GZfos18C8	Archea	357/38.1	FKBP<br>Cyclophilin
*Ar*FCBP39.7	CAI64202	uncultured archaeon GZfos18C8	Archea	369/39.7	FKBP<br>Cyclophilin
*Cb*CypB	ZP_00909097	*Clostridium beijerincki*	Bacteria, Clostridia	226/24.1	Cyclophilin
*Mb*Cyp15.3	YP_566142	*Methanococcoides burtonii*	Archaea, Methanomicrobia	141/15.3	Cyclophilin
*Gf*CFBP41	CAL65762	*Gramella forsetii*	Flavobacteria	376/41.0	Cyclophilin<br>FKBP
*Ca*CFBP41.7	ZP_00950792	*Croceibacter atlanticus*	Flavobacteria	378/41.7	Cyclophilin<br>FKBP
*Fb*CFBP42.3	ZP_01106954	*Flavobacteriales bacterium*	Flavobacteria	386/42.3	Cyclophilin<br>FKBP
*Fba*CFBP42.1	ZP_01202369	*Flavobacteria bacterium*	Flavobacteria	385/42.1	Cyclophilin<br>FKBP
*Cs*CFBP42.8	ZP_01051599	*Cellulophaga sp.* MED134	Flavobacteria	390/42.8	Cyclophilin<br>FKBP
*Lb*CFBP44.4	ZP_01061314	*Leeuwenhoekiella blandensis*	Flavobacteria	392/44.4	Cyclophilin<br>FKBP
*Dp*CFBP36.7	YP_065381	*Desulfotalea psychrophila*	Proteobacteria	344/36.7	Cyclophilin<br>FKBP

Table S2: Protein sequences used to analyse evolution of cyclophilin domains in dual class immunophilins (continued)

Name	Accession-no.	Species	Group	Size (amino acids/kDa)	Domains[a]
*Td*CFBP35.5	NP_972990	*Treponema denticola* ATCC 35405	Spirochaetes	322/35.5	Cyclophilin FKBP
*Bh*CFBP38	YP_001883841	*Borrelia hermsii* DAH	Spirochaetes	339/38	Cyclophilin FKBP
*Hm*Cyp22.7	YP_137701	*Haloarcula marismortui*	Archaea, Halobacteria	209/22.7	Single cyclophilin
*Hw*Cyp19.7	YP_658820	*Haloquadratum walsbyi*	Archaea, Halobacteria	179/19.7	Single cyclophilin
*Mg*Cyp17.2	XP_366921	*Magnaporthe grisea*	Fungi	159/17.2	Single cyclophilin
*Pn*Cyp18	EAT84755	*Phaeosphaeria nodorum*	Fungi	164/18.0	Single cyclophilin
*Aa*Cyp19.4	EAT41295	*Aedes aegypti*	Insecta	176/19.4	Single cyclophilin

Table S3: Protein sequences used to analyse evolution of FKBP domains in dual class immunophilins

Name	Accession-no.	Species	Group	Size (amino acids/kDa)	Domains[a]
*Pt*FCBP51.3	XP_001444554	*Paramecium tetraurelia* strain d4-2	Ciliophora	456/51.3	FKBP<br>TPR repeats<br>cyclophilin
*Pt*FCBP52.6	XP_001445268	*Paramecium tetraurelia* strain d4-2	Ciliophora	467/52.6	FKBP<br>TPR repeats<br>cyclophilin
*Tg*FCBP57.3	AAX51680	*Toxoplasma gondii*	Apicomplexa	521/57.3	FKBP<br>TPR repeats<br>cyclophilin
*Lm*FKBP47.7	CAJ07250	*Leishmania major*	Kinetoplastida	432/47.7	FKBP<br>TRP
*Tn*FKBP34.2	CAG13057	*Tetraodon nigroviridis*	Vertebrata	303/34.2	FKBP<br>TRP
*Pca*FCBP52.5	BT031867	*Phytophora capsici*	Oomycetes	479/52.5	FKBP<br>TPR repeats<br>cyclophilin
*Tt*FCBP131.6	XP_976669	*Tetrahymena thermophila*	Ciliophora	1134/131.6	ATB-binding<br>FKBP<br>TRP<br>cyclophilin
*Ta*FCBP51.3	XP_951815[b]	*Theileria annulata*	Apicomplexa	459/51.3[b]	FKBP<br>TPR repeats<br>cyclophilin
*Bb*FCBP51.1	YP_001883841	*Babesia bovis*	Apicomplexa	339/51.1	FKBP<br>TPR repeats<br>cyclophilin
*Tp*FCBP51.4	XP_765469	*Theileria parva*	Apicomplexa	460/51.4	FKBP<br>TPR repeats<br>cyclophilin
*Py*FKBP35	XP_730233	*Plasmodium yoelii*	Apicomplexa	306/35.0	FKBP<br>TRP
*Pc*FKBP31	CAH81605	*Plasmodium chabaudi*	Apicomplexa	271/31.0	FKBP<br>TRP
*Pb*FKBP33.1	XP_672280	*Plasmodium berghei*	Apicomplexa	289/33.1	FKBP<br>TRP
*Pf*FKBP34.8	NP_701815	*Plasmodium falciparum*	Apicomplexa	304/34.8	FKBP<br>TRP
*Ec*FKBP12.3[c]	CAB57241	*Entodinium caudatum*	Ciliophora	113/12.3	Single FKBP
*Ss*FKBP21.6	NP_440378	*Synechocystis sp.* PCC 6803	Cyanobacteria	201/21.6	Single FKBP
*Te*FKBP21.6	YP_721230	*Trichodesmium erythraeum*	Cyanobacteria	203/21.6	Single FKBP
*Td*CFBP35.5	NP_972990	*Treponema denticola* ATCC 35405	Spirochaetes	322/35.5	Cyclophilin<br>FKBP
*Cs*CFBP42.8	ZP_01051599	*Cellulophaga sp.* MED134	Flavobacteria	390/42.8	Cyclophilin<br>FKBP
*Lb*CFBP44.4	ZP_01061314	*Leeuwenhoekiella blandensis*	Flavobacteria	392/44.4	Cyclophilin<br>FKBP
*Gf*CFBP41	CAL65762	*Gramella forsetii*	Flavobacteria	376/41.0	Cyclophilin<br>FKBP
*Fb*CFBP42.3	ZP_01106954	*Flavobacteriales bacterium*	Flavobacteria	386/42.3	Cyclophilin<br>FKBP
*Fb*CFBP42.1	ZP_01202369	*Flavobacteria bacterium*	Flavobacteria	385/42.1	Cyclophilin<br>FKBP
*Ca*CFBP41.7	ZP_00950792	*Croceibacter atlanticus*	Flavobacteria	378/41.7	Cyclophilin<br>FKBP
*Tc*MIP	Q09734	*Trypanosoma cruzi*	Kinetoplastida	192/22.1	Single FKBP
*No*FKBP22.1	YP_343250	*Nitrosococcus oceani*	Proteobacteria	225/24.9	FKBP_N<br>FKBP
*Ps*FKBP14.5	YP_548695	*Polaromonas sp.* JS666	Proteobacteria	140/14.5	Single FKBP
*Dp*CFBP36.7	YP_065381	*Desulfotalea psychrophila*	Proteobacteria	344/36.7	Cyclophilin<br>FKBP
*Yl*FKBP15.6	XP_500249	*Yarrowia lipolytica*	Fungi	144/15.6	Single FKBP
*Bh*CFBP38	YP_001883841	*Borrelia hermsii* DAH	Spirochaetes	339/38	Cyclophilin<br>FKBP
*Gs*FKBP16.3	ZP_01389980	*Geobacter sp.* FRC-32	Proteobacteria	150/16.3	Single FKBP
*Ar*FCBP38.1	AAU82569	uncultured archaeon GZfos18C8	Archea	357/38.1	FKBP<br>Cyclophilin
*Ar*FCBP39.7	CAI64202	uncultured archaeon GZfos18C8	Archea	369/39.7	FKBP<br>Cyclophilin
*Mba*FKBP18.8	YP_305460	*Methanosarcina barkeri*	Archea, Methanomicrobia	169/18.8	Single FKBP

Table S3: Protein sequences used to analyse evolution of FKBP domains in dual class immunophilins (continued)

Name	Accession-no.	Species	Group	Size (amino acids/kDa)	Domains[a]
*Mbu*FKBP16.6	YP_566866	*Methanococcoides burtonii*	Archea, Methanomicrobia	152/16.6	Single FKBP
*Mt*FKBP17.2	O52980	*Methanothermococcus thermolithotrophicus*	Archea, Methanococci	154/17.2	Single FKBP
*Mba*FKBP20.6	YP_305459	*Methanosarcina barkeri*	Archea, Methanomicrobia	189/20.6	Single FKBP
*Ma*FKBP19.4	NP_618030	*Methanosarcina acetivorans*	Archea, Methanomicrobia	181/19.4	Single FKBP
*Mma*FKBP17.5	ZP_01392854	*Methanoculleus marisnigri*	Archea, Methanomicrobia	167/17.5	Single FKBP
*Ot*CPR7	CAL55134	*Ostreococcus tauri*	Chlorophyta	>266 (only partial sequence)	FCBP cyclophilin
*Cf*FKBP15.4	ZP_01386099	*Chlorobium ferrooxidans*	Bacteria, Chlorobia	142/15.4	Single FKBP
