# Supplementary material for: In silico analysis of the cyclophilin repertoire of apicomplexan parasites
Source: Parasit Vectors. 2009 Jun 25;2:27. doi: 10.1186/1756-3305-2-27 (PMC2713222; doi:10.1186/1756-3305-2-27)
Supplement: Additional file 5 — Figure S3 – FCBP and CFBP proteins in non-apicomplexa. Domain architecture of FCBPs and CFBPs from non-apicomplexan organisms. [file 1756-3305-2-27-S5.pdf]

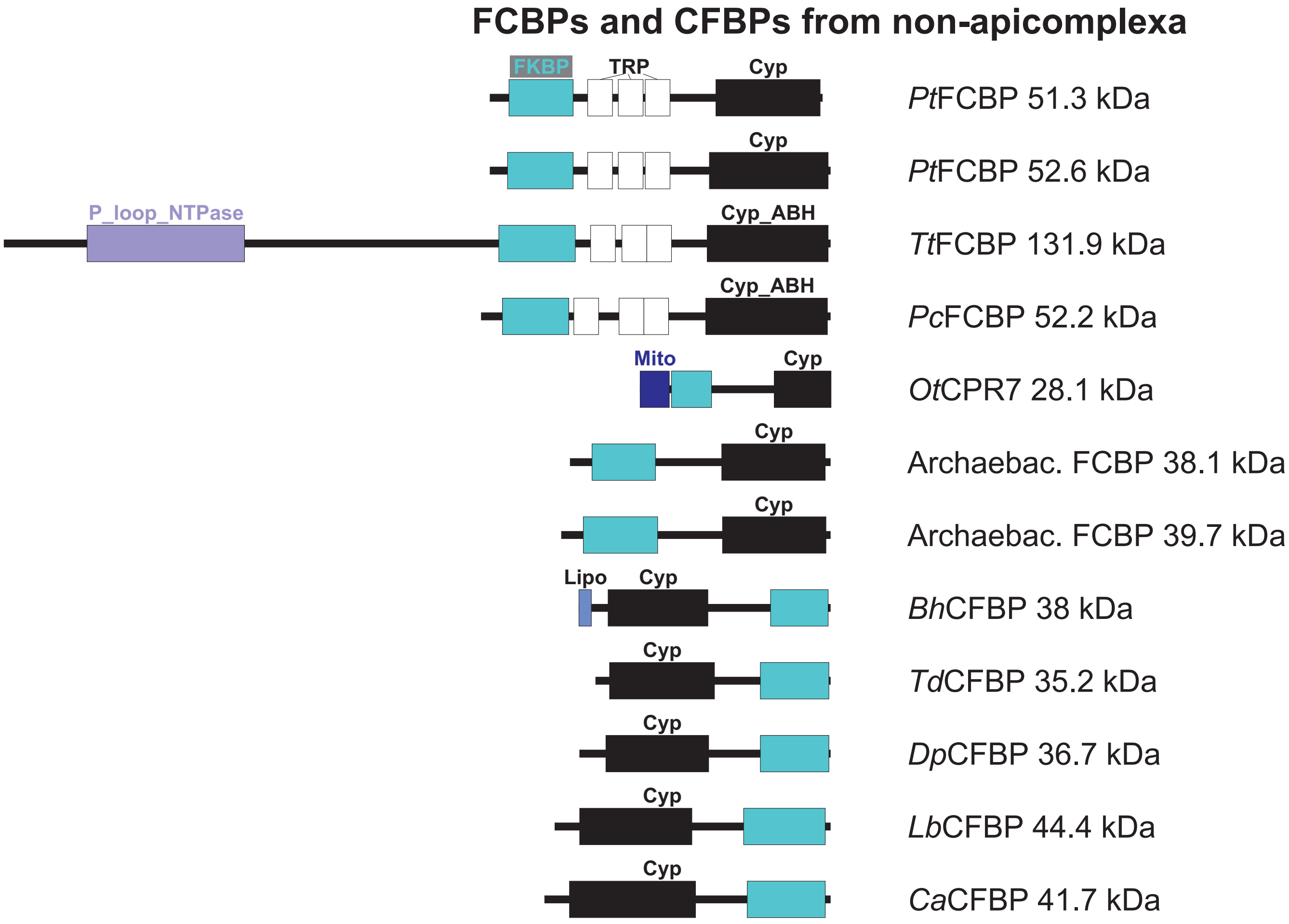

Figure S3 - FCBP and CFBP proteins in non-apicomplexa

Domain architecture of FCBPs and CFBPs from non-apicomplexan organisms. Species are abbreviated as in Fig. 1. Cyp_ABH, ABH-type Cyp domain (CD accession-no.: cd01926); FKBP, FK506-binding domain (PFAM accession-no.: pfam00254); TPR, Tetratricopeptide repeat (InterProScan accession-no.: [IPR001440]); Mito, mitochondrial localization signal; Lipo, bacterial lipoprotein anchor sequence (PROSITE accession-no.: [PS51257]); P-loop NTPase, P-loop containing Nucleoside Triphosphate Hydrolases (CD accession-no.: [cl09099]).
