# Supplementary material for: In silico analysis of the cyclophilin repertoire of apicomplexan parasites
Source: Parasit Vectors. 2009 Jun 25;2:27. doi: 10.1186/1756-3305-2-27 (PMC2713222; doi:10.1186/1756-3305-2-27)
Supplement: Additional file 6 — Figure S4 – PPIL1-like Cyps. Domain architecture and genomic organization of PPIL1-like Cyps. [file 1756-3305-2-27-S6.pdf]

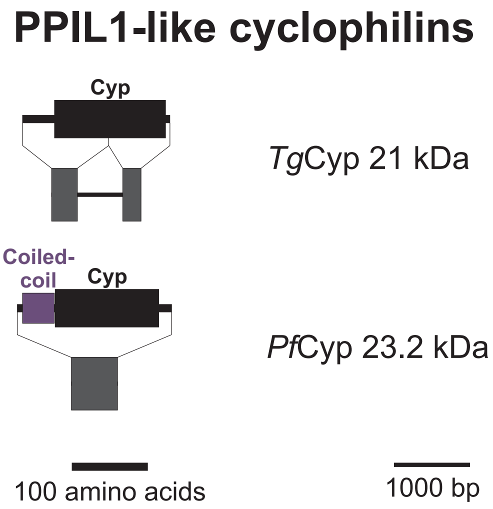

Figure S4 - PPIL1-like Cyps
Domain architecture and genomic organization of PPIL1-like Cyps. Species are abbreviated as in Fig. 1. Cyp, domain belonging to the Cyp superfamily (CD accession-no: [cl00197]); Coiled-coil, coiled-coil protein interaction region.
