# Supplementary material for: In silico analysis of the cyclophilin repertoire of apicomplexan parasites
Source: Parasit Vectors. 2009 Jun 25;2:27. doi: 10.1186/1756-3305-2-27 (PMC2713222; doi:10.1186/1756-3305-2-27)
Supplement: Additional file 7 — Figure S5 – PPIL4-like Cyps. Domain architecture and genomic organization of ChCyp34.5, the only apicomplexan PPIL4-like Cyp which contains an RNA recognition motif. [file 1756-3305-2-27-S7.pdf]

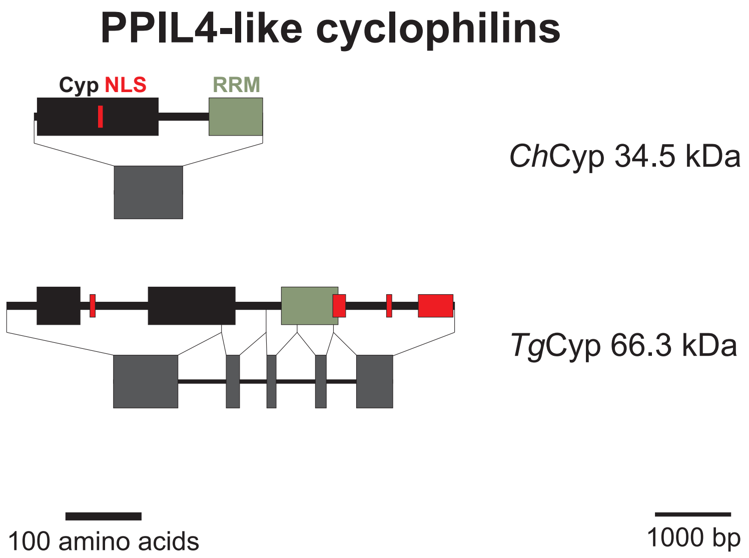

Figure S5 - PPIL4-like Cyps

Domain architecture and genomic organization of *Ch*Cyp34.5, the only apicomplexan PPIL4-like Cyp which contains an RNA recognition motif. Species are abbreviated as in Fig. 1. Cyp, Cyp superfamily domain (CD accession-no.: [cl00197]); NLS, nuclear localization signal; RRM, RNA recognition motif (CD accession-no.: cd00590).
